# Sweet Taste Preference: Relationships with Other Tastes, Liking for Sugary Foods and Exploratory Genome-Wide Association Analysis in Subjects with Metabolic Syndrome

**DOI:** 10.3390/biomedicines10010079

**Published:** 2021-12-31

**Authors:** Rebeca Fernández-Carrión, Jose V. Sorlí, Oscar Coltell, Eva C. Pascual, Carolina Ortega-Azorín, Rocío Barragán, Ignacio M. Giménez-Alba, Andrea Alvarez-Sala, Montserrat Fitó, Jose M. Ordovas, Dolores Corella

**Affiliations:** 1 Department of Preventive Medicine and Public Health, School of Medicine, University of Valencia, 46010 Valencia, Spain; rebeca.fernandez@uv.es (R.F.-C.); jose.sorli@uv.es (J.V.S.); pascaseva89@gmail.com (E.C.P.); carolina.ortega@uv.es (C.O.-A.); rocio.barragan@uv.es (R.B.); i.gimenez.alba@uv.es (I.M.G.-A.); andrea.alvarez@uv.es (A.A.-S.); 2 CIBER Fisiopatología de la Obesidad y Nutrición, Instituto de Salud Carlos III, 28029 Madrid, Spain; oscar.coltell@uji.es (O.C.); mfito@imim.es (M.F.); 3 Department of Computer Languages and Systems, Universitat Jaume I, 12071 Castellon, Spain; 4 Sleep Center of Excellence, Department of Medicine, Columbia University Irving Medical Center, New York, NY 10032, USA; 5 Division of General Medicine, Department of Medicine, Columbia University Irving Medical Center, New York, NY 10032, USA; 6 Cardiovascular Risk and Nutrition Research Group (CARIN), Hospital del Mar Research Institute (IMIM), 08003 Barcelona, Spain; 7 Nutrition and Genomics Laboratory, JM-USDA Human Nutrition Research Center on Aging at Tufts University, Boston, MA 02111, USA; jose.ordovas@tufts.edu; 8 Nutritional Genomics and Epigenomics Group, IMDEA Alimentación, 28049 Madrid, Spain

**Keywords:** biomedicine, personalized nutrition, sweet taste preference, taste perception, sugary foods, type 2 diabetes, GWAS, genetic markers

## Abstract

Taste perception and its association with nutrition and related diseases (type 2 diabetes, obesity, metabolic syndrome, cardiovascular, etc.) are emerging fields of biomedicine. There is currently great interest in investigating the environmental and genetic factors that influence sweet taste and sugary food preferences for personalized nutrition. Our aims were: (1) to carry out an integrated analysis of the influence of sweet taste preference (both in isolation and in the context of other tastes) on the preference for sugary foods and its modulation by type 2 diabetes status; (2) as well as to explore new genetic factors associated with sweet taste preference. We studied 425 elderly white European subjects with metabolic syndrome and analyzed taste preference, taste perception, sugary-foods liking, biochemical and genetic markers. We found that type 2 diabetic subjects (38%) have a small, but statistically higher preference for sweet taste (*p* = 0.021) than non-diabetic subjects. No statistically significant differences (*p* > 0.05) in preferences for the other tastes (bitter, salty, sour or umami) were detected. For taste perception, type 2 diabetic subjects have a slightly lower perception of all tastes (*p* = 0.026 for the combined “total taste score”), bitter taste being statistically lower (*p* = 0.023). We also carried out a principal component analysis (PCA), to identify latent variables related to preferences for the five tastes. We identified two factors with eigenvalues >1. Factor 2 was the one with the highest correlation with sweet taste preference. Sweet taste preference was strongly associated with a liking for sugary foods. In the exploratory SNP-based genome-wide association study (GWAS), we identified some SNPs associated with sweet taste preference, both at the suggestive and at the genome-wide level, especially a lead SNP in the PTPRN2 (Protein Tyrosine Phosphatase Receptor Type N2) gene, whose minor allele was associated with a lower sweet taste preference. The PTPRN2 gene was also a top-ranked gene obtained in the gene-based exploratory GWAS analysis. In conclusion, sweet taste preference was strongly associated with sugary food liking in this population. Our exploratory GWAS identified an interesting candidate gene related with sweet taste preference, but more studies in other populations are required for personalized nutrition.

## 1. Introduction

In many studies, the dietary intake of sugary foods has been associated with an increased risk of obesity, type 2 diabetes, metabolic syndrome, and other diseases [1,2,3]. For personalized nutrition (based on the notion that individualizing dietary advice will be more effective than general approaches), there is, therefore, a growing interest in understanding the factors that influence why some people consume more sugary foods in their diet. Precision nutrition has been promoted by the National Institutes of Health (NIH) within the current Plan for NIH Nutrition Research [4], but results are still in their infancy and more research is needed [5]. Although the factors that contribute to a higher intake of sweet foods are many and varied, it is thought that taste may be an important determinant [6,7,8,9]. In several studies undertaken on populations of different ages, participants stated that food taste was one of the factors that most influenced their intake [10,11,12,13,14,15,16]. In general, people state that they prefer to eat what they like, and sweet is one of the most preferred tastes [17,18,19,20]. This can be explained by the fact that, in terms of the survival of the species, sweet taste used to be associated with nutritious foods, rich in energy, conferring an advantage in environments of scarcity [21]. Unlike bitter taste, which was generally associated with possibly toxic foods, sweet taste is preferred in childhood and provides a feeling of safety when ingested [22]. Furthermore, there is an innate basis for the sensory pleasure derived from tasting sweet products [20].

Despite there being a growing consensus on the influence of taste in selecting foods consumed [23,24,25,26,27], there are still many doubts about how this influence is exerted. Many studies have analyzed sweet taste perception (including a range of measurement (i.e., threshold testing or sweet taste intensity) and have related it to food intake or to obesity-related variables [9,18,24,28,29,30]. However, the studies analyzing the association between sweet taste perception and food intake have reported inconsistent results. Another relevant issue is to measure the preference for sweet taste [9,23]. It has been reported that, in general, sweet taste preference is more likely to be associated with dietary intake than sweet taste perception [9], despite the degree of heterogeneity among the methods to evaluate sweet taste preference [19,20,21,22,23,24]. Moreover, the relationship between the perception score for a taste quality and the preference for it is not clear. Some authors claim that the greater the ability to perceive sweet taste, the lower the preference for it, resulting in a lower intake of sweet foods [24,29]. However, few studies have analyzed both the ability to perceive sweet taste and its preference. In addition, longitudinal changes in sweet taste preferences in humans have been described [18,31] (in general indicating that children and adolescents preferred higher sucrose concentrations than adults).

On the other hand, it is known that there is an important genetic influence on taste perception [30,32,33,34]. Several polymorphisms in taste receptors, and in genes related to peripheral taste transduction and central taste processing have been reported [30,32,35,36]. Although taste receptors for sweet and umami [T1R (taste receptor, type 1)] as well as for bitter [T2R (taste receptor, type 2)] have been characterized in humans [32,37], the genetic influence at the population level, is especially well known for bitter taste, where common polymorphisms in the TAS2R38 (taste 2 receptor member 38) gene have been strongly associated with bitter taste perception [35]. However, for the perception of the other tastes (sweet, salty, sour and umami), the genetic influence at the population levels is less well understood. For sweet taste, the first sweet-related subunit cloned was T1R2, and later, the T1R3 subunit was identified [30]. Further, several sweet taste receptor independent pathways have been characterized [30,32,36,37]. Based on the candidate gene approach, polymorphisms in several sweet taste-related genes (TAS1R1, TAS1R2, TAS1R3, GRM1, SLC2A2, DRD2, OPRM1, SCNN1A, SCNN1B, SCNN1G, SCNN1D, TRPV1, and GNAT3, among others) have been associated with sweet taste perception or with sweet taste preferences in some studies, but consistency among investigations is very low [22,32,33,34,36,37]. In the genome-wide era, some initial Genome-Wide Association Studies (GWASs), aimed at identifying novel genes related to taste perception, were undertaken mainly for bitter taste [32,38,39,40,41]. These GWASs consistently identified SNPs in the TAS2R38 gene as strongly associated with bitter taste perception [38,39,41], but very few studies, focusing on GWASs for sweet taste perception [40,41] have been undertaken, apart from our previous GWAS for sweet taste perception in Mediterranean subjects [41].

Furthermore, less well known is the genetic influence on taste preferences [32,42,43] at the GWAS level. Although some recent GWASs have been published on sweet taste preferences and preference for sugary foods intake [40,44,45,46], in general, we can only say that the genes determining preference for sweet taste are very little known. One of the factors contributing to this lack of knowledge is that very few of these cohorts have measured sweet taste preference [40,45], before carrying out GWAS on the consumption of sugary foods, as a proxy for sweet taste preference. Even when sweet taste preference has been measured in the mentioned GWAS, heterogeneity in this phenotype has been detected: Hwang et al. [40] analyzed sucrose liking in 686 individuals (twins and families from the United States), whereas Kawafune et al. [45] obtained sweet taste preference from more than 12,000 Japanese by using an internet-based questionnaire, resulting in a five-point scale of preference. Using food consumption as a proxy of the sweet taste preference, Hwang et al. [40] analyzed the intake of sugars and sweets in Europeans; Suzuki et al. [43] focused on the intake of sweets in Japanese; and Zhong et al. [46] analyzed sweet-tasting beverages in European subjects from the UK Biobank. Considering the heterogeneity of the phenotypes analyzed, the inconsistency of the results obtained in the published GWAS is not surprising. Therefore, more GWAS analyzing a measured sweet taste preference phenotype, instead of using food intake as a proxy, are needed.

Further, for precision medicine, it is very important to consider the population characteristics. Genetic ancestry matters in precision medicine and currently, there is a scarcity of GWASs carried out in European Mediterranean populations, which for some markers differ from Northern and Central European populations [47]. In addition to the genetic background, other individual characteristics such as sex, age, presence of cardiovascular risk factors such as metabolic syndrome or type 2 diabetes, have emerged as very relevant factors for personalized nutrition [4,5]. Regarding sweet taste in type 2 diabetes, [48,49], although some studies suggested that type 2 diabetic subjects may show poor taste sensation [50,51,52,53] mostly for sweet taste perception, so increasing the search for foods richer in sugars, there is still a lack of consistency [48,49,50,51,52,53] and it is necessary to delve deeper into this issue. Lastly, another factor to be considered is the fact that tastes have mostly been analyzed separately [32] when the greater or lesser preference for one taste quality possibly influences the preference for another, and this should be investigated. Therefore, our aims were as follows: (1) To analyze preference for sweet taste and its relationship with the preference for and perception of other tastes, identifying combined sweet-taste factors, and then examining the association between sweet taste preferences and sugary foods in elderly Mediterranean subjects with metabolic syndrome, taking into account the influence of type 2 diabetes; and (2) To explore the genes and genetic variants most associated with preference for sweet taste in this Mediterranean population by both using SNP-based and gene-based GWAS approaches.

## 2. Materials and Methods

### 2.1. Study Design and Participants

We have carried out a cross-sectional study in 425 white Caucasian subjects with metabolic syndrome, aged 55 to 75 years, from a European Mediterranean population. These individuals are participants in the PREDIMED Plus-Valencia study, one of the field centers of the ongoing multi-center PREDIMED Plus trial [54]. The participants in this study were recruited in the various primary health care centers of the Valencia region. This region is located on the eastern Mediterranean coast of Spain. Participants were community-dwelling adults (men, 55–75 years; women, 60–75 years) with a body-mass index (BMI) in the overweight or obesity range [(BMI) ≥ 27 and <40 kg/m^2^] who, at baseline, met at least three components of metabolic syndrome [55]. Some of these participants (38%) also presented type 2 diabetes [54]. Although the total number of participants recruited in the Valencia field center was 465 at baseline, we only included the data from 425 subjects in this study, as this was the number of subjects who agreed to participate in the sub-study of taste preferences and who also completed the questionnaires on taste preferences and food preferences (see Figure S1 for the graphic diagram representing the study design, including the sequences of experiments and statistical analysis). At baseline, these participants did not differ significantly from our entire sample concerning the main variables. Participants provided written informed consent and study protocols and procedures were approved according to the ethical standards of the Helsinki Declaration and by the Human Research Ethics Committee of Valencia University, Valencia (ethical approval code H1373255532771, 15 July 2013; and H1509263926814, 6 November 2017).

### 2.2. Baseline Anthropometric, Clinical and Biochemical Variables

Anthropometric variables and blood pressure were determined at baseline by trained staff and following the PREDIMED-Plus operations protocol [54,55]. Weight and height were measured with calibrated scales on a wall-mounted stadiometer, respectively. BMI was calculated as the weight in kilograms divided by height in meters squared. Waist circumference was measured midway between the lowest rib and the iliac crest, after normal exhalation, using an anthropometric tape. Blood pressure was measured using a validated semiautomatic oscillometer (Omron HEM-705CP, Netherlands) while the participant was in a seated position after 5 min of rest [55]. Blood samples were collected after a 12-h overnight fast. Fasting plasma glucose, total cholesterol, HDL-C and triglyceride concentrations were measured in a clinical laboratory using standard enzymatic methods and LDL-C concentrations were estimated with the Friedewald formula [54]. Medication use was measured at baseline using a validated questionnaire [55]. Type 2 diabetes was defined as a previous clinical diagnosis of type 2 diabetes or HbA1c levels ≥ 6.5% [55]. Obesity was defined as having a BMI ≥ 30 kg/m^2^.

### 2.3. Baseline Lifestyle Variables and Adherence to the Mediterranean Diet

Tobacco smoking was measured as previously reported [54,55]. Leisure-time physical activity was assessed using the validated REGICOR questionnaire. Physical activity-related energy expenditure was estimated as the summed product of frequency, duration, and intensity of each activity divided by 30 days/month (MET·min/day). A 17-item screening questionnaire was used for assessing adherence to an energy-restricted Mediterranean diet [56]. Table S1 shows the detailed questions included in the 17-item questionnaire. For this study, focusing specifically on the influence of sweet taste, we selected the following items from the questionnaire’s 17 items for a more in-depth study, corresponding to sugary foods: “item 6: How many sugar-sweetened beverages (soft drinks, cola, bitter, juices) do you drink per week?”; “item 9: How many times per week do you consume pastries such as cookies, sweets or cakes?”, and “item 13: Do you add sugar to your beverages (coffee, tea)?” We made no in-depth analysis of the other items on the scale, given that carrying out a detailed study of the association between sweet taste preferences and the profile of foods and nutrients consumed was not the aim of this study and that that analysis would require a deeper investigation and will be the object of another study.

### 2.4. Taste Preferences and Food Preferences

The preference for different tastes (sweet, salty, bitter, sour and umami) was measured in the same way for all five tastes. The 9-point hedonic scale [57] was used. This scale has nine categories, and each category is associated with a verbal descriptor from “Dislike extremely” (value 1) to “Like extremely” (value 9). The scale also has a neutral category “neither like nor dislike”. The reliability, validity, and discriminative ability of the 9-point hedonic scale have been demonstrated [57]. No stimuli for taste preference were administered. Trained personnel staff provided a detailed explanation of the use of the scale. For the food liking questionnaire, and bearing in mind that this was an elderly population and that the number of foods to be appraised was higher (37-items), a scale with a lower number of points (response categories) was preferred so as to simplify the selection process [58]. We used a simpler 4-point Likert-scale, by adapting the 9-point scale of previous food linking questionnaires [59]. The mid-point category (neither like nor dislike) was removed to minimize the neutral position bias [60]. Participants scored on a rising scale of food preferences from 1-“extreme dislike” to 4-“extreme like”. The foods included in the food liking questionnaire were adapted to the characteristics of this population, including: whole milk, semi/skimmed milk, whole yogurt, semi/skimmed yogurt, eggs, red meat, poultry, blue fish, white fish, mature cheeses, fresh cheese, cold meats, bread, pasta, legumes, green beans, broccoli, artichokes, oranges, lemons, other fruits, olive oil, sunflower oil, other oils, butter, margarine, aioli, salt-cured foods, spicy foods, spices, nuts, breakfast cereals, sweets and ice cream, chocolates, and sugar. In this study, however, we only focus on sugary foods, including “breakfast cereals”, “sweets-pastries and ice creams”, “chocolates” and “sugar”. The preference for these foods has been analyzed both individually and by calculating the combined scores of the foods considered.

### 2.5. Taste Perception Tests

Of the 425 subjects who completed the taste and food preference questionnaires, baseline data on the perception of five tastes was available from 348 of them. The methodology for carrying out the taste perception tests has already been described in a previous publication [41]. Briefly, each individual was exposed to five concentrations (concentrations I, II, III, IV and V) for each of the five tastes (bitter, sweet, salty, sour and umami). Trained staff provided a detailed explanation of the procedures prior to starting the series of tests. A single tastant was used for sweet, salty, sour and umami; sucrose, NaCl, citric acid and L-glutamic acid monopotassium salt monohydrate (MPG), respectively. For bitter taste, two tastants were used; phenylthiocarbamide (PTC) and 6-n-propylthiouracil (PROP) (all from Sigma-Aldrich, Milan, Italy). Distilled water was used as the solvent. Five solutions of different concentrations (I to V) were prepared for each tastant, including a distilled-water control. The series of concentrations (I to V) used for each tastant has already been reported in detail [41], as well as the way of administering the tastants and carrying out the tests [41,61]. Briefly: The series of concentrations (I to V) used were as follows: for sucrose 100 mM, 150 mM, 200 mM, 300 mM and 400 mM; for NaCl 25 mM, 50 mM, 75 mM, 100 mM and 200 mM; for citric acid 1 mM, 5 mM, 10 mM, 17mM and 34 mM; for MPG 25 mM, 50 mM, 75 mM, 100 mM and 200 mM; for PTC 0.056 mM, 0.18 mM, 0.56 mM, 1.8 mM and 5.6 mM; and for PROP 0.055, 0.17, 0.55, 1.7, and 5.5 mM [41]. The tests for bitter taste, both PTC and PROP, were undertaken on strips of filter paper and presented in pre-set defined random order (the same for each participant). The other tastants were prepared and tested in liquid form, dissolved to the concentration indicated, and were presented in different small colored tubes for each taste and organized into racks of a pre-set order (increasing concentrations) and the same for all participants. Before beginning the taste perception tests, participants had to rinse their mouths several times with spring water. The order in which the tests for the different tastants were conducted was the same for each participant. Tastants were presented in blocks of taste starting with sweet, then salty, sour, umami and bitter. Subjects assessed the intensity of tastants on a scale. All participants were given a template on which they had to complete the scale of taste perception intensity for each taste and concentration (participants only saw the tube or the ordered filter paper marked with a symbol). Between each set of major taste challenges there was a (3–5)-min break. Although the general labeled magnitude scale (gLMS) has been proposed in general as a good standard for intensity rating [29,32], in our study we have used the category scale, bearing in mind the difficulty that older subjects experience in understanding the logarithmic LMS and the need to use a simplified scale in this group [41]. Thus, we used a vertical scale consisting of 6 intensity values from 0 (bottom) to 5 (top). The labels used for these values were: 0 meaning “no taste”, 1 “weak”, 2 “moderate”, 3 “strong”, 4 “very strong” and 5 “extremely strong”. The same scoring scale was used for all tastes. Although we obtained taste scores for all five concentrations and for PTC and PROP for bitter, in this study, we only used the scores obtained for the highest concentration of each tastant (concentration V) for the statistical analyses, and selected PTC for bitter, as previously reported [41]. Thus, the tastants (including a blank for each tastant) and concentrations specifically used in this paper were: 5.6 mM for PTC for bitter; 400 mM for sucrose; 200 mM for NaCl and MPG; and 34 mM for citric acid, in order to maximize the differences in intensity rating between individuals. With these scores for the five taste qualities, we constructed a “total taste score” summing up the points obtained for each of the individual tastes for the corresponding concentration tested (concentrations V). The range of the total taste score was from 0 to 25 points [41]. A higher score indicates higher taste perception.

### 2.6. DNA Isolation and Genome-Wide Genotyping

Genomic DNA was isolated from blood. The quantity of double-stranded DNA was measured using PicoGreen (Invitrogen Corporation, Carlsbad, CA, USA) and 415 DNA-samples were optimal. High-density genotyping was performed at the University of Valencia using the Infinium OmniExpress-24 v1.2 BeadChip genotyping array, capturing 713,599 markers, (Illumina Inc., San Diego, CA, USA) according to the manufacturer’s protocol with appropriate quality standards as previously reported [54]. Allele detection and genotype calling were performed in the Genome Studio genotyping module (Illumina, Inc.). Data cleaning was performed using standard analysis pipelines implemented in the Phyton programming language using the Numpy library modules combined with the PLINK [62,63]. SNPs not mapped on autosomal chromosomes were filtered out. In addition, SNPs with a minor allele frequency (MAF) < 0.01, those that deviated from expected Hardy-Weinberg equilibrium (*p* < 1.0 × 10^−4^), and SNPs with a low call-rate (<90%) were removed. The overall call-rate in these subjects exceeds 99% genotyping. Using genome-wide genotyping we undertook various exploratory GWASs to identify which gene variants are most associated with sweet taste preference. To do so, we used two types of analysis. Firstly, we used the classic GWAS based on analyzing SNPs [54], and secondly, we undertook an exploratory GWAS using the gene-based analysis approach [64,65]. In the following statistical analysis, we provide more details.

### 2.7. Statistical Analysis

First of all, we carried out descriptive statistical tests to summarize the characteristics of the sample studied. Chi-square tests were used to compare proportions. Student *t*-tests and ANOVA tests were applied to compare crude means of continuous variables. Triglyceride concentrations were log-transformed for the statistical analyses. Spearman’s rank correlation coefficients were calculated to estimate the association between preference for sweet taste and preferences for the other tastes. Spearman correlation coefficients were also used to calculate the association between taste preferences and taste perception. General linear models adjusted for potential confounders were used to estimate the associations between taste preferences and sugary foods (preferences and intake). Models were sequentially fitted as follows: model 1, unadjusted; model 2, adjusted for age and sex; model 3, additionally adjusted for type 2 diabetes. Additional adjustments for BMI, smoking, physical activity, hours of sleep per night and adherence to the Mediterranean diet were carried out when indicated. Adjusted means for continuous variables were estimated from the multivariate-adjusted general linear models. In some analyses, taste preferences or food preferences were dichotomized (depending on the population mean) and associations with the corresponding variables were estimated by logistic regression models. Odds ratios (OR) and 95% confidence intervals (CI) for the corresponding variables were estimated in logistic regression models. Logistic regression models were also multivariable-adjusted as indicated above for the linear models. Analyses were undertaken for the whole population and stratified by type 2 diabetes when indicated.

We also carried out a factor analysis with the five taste preference variables (sweet, bitter, salty, sour and umami) to identify the latent multidimensionality by identifying a potentially lower number of unobserved (latent) variables called factors. The appropriateness of this analysis was evaluated based on the Kaiser-Meyer-Olkin (KMO) value and Bartlett’s test (homogeneity of variance). Principal component analysis (PCA) was used for extracting factors. We used the Kaiser criterion (components that have eigenvalues greater than 1) for selecting the optimal number of components in the factor analysis. We used orthogonal rotation (varimax) to clarify the factors. The varimax method attempts to minimize the number of high loading variables on one latent factor [66]. For each individual, the scores of the factors obtained were calculated and these factor scores were later used as latent variables of combined taste preferences in the corresponding association analyses.

In the specific study on the association between taste preferences and preference of sweet foods or consumption of sugary foods included in the adherence to the Mediterranean diet scale, sweet taste preference was used as the original variable (collapsed as dichotomous). These statistical analyses were performed with the IBM SPSS Statistics version 26.0, NY. All tests were two-tailed and *p* values < 0.05 were considered statistically significant for these associations.

To identify the genes that are associated with the greater or lesser preference for sweet taste, we undertook an exploratory GWAS analysis using PLINK v1.9 [62,63]. We considered 2 approaches: A classical SNP-based GWAS, as well as a novel gene-based GWAS [64,65]. The SNP-based GWAS was carried out for the main variable of this study, which is the measured sweet taste preference. This variable was considered firstly as a dichotomous variable for the GWAS association analysis. An exploratory analysis was also conducted for the sweet taste preference as a continuous variable, with the sole aim of checking the consistency of the results. Secondly, we used the latent variable for the combined sweet taste factor (as a continuous variable) obtained in the PCA for GWAS.

The alleles for each SNP were considered in accordance with an additive model (0, 1, or 2 copies of the variant allele). When the dependent variable was considered continuous, general linear models were fitted, both unadjusted and multivariable-adjusted, including the different confounding variables (sex, age, type 2 diabetes, as specifically indicated in each model). Regression coefficients for the minor allele were estimated. Likewise, when the dependent variable was dichotomous (greater or lesser preference for sweet taste) we used logistic regression analysis (unadjusted and adjusted for the indicated potential confounders). For the SNP-based GWAS, we used the conventional threshold of *p* < 5 × 10^−8^ for genome-wide statistical significance. Likewise, according to the conventional GWAS rules, SNPs with *p*-values below 1 × 10^−5^ were also considered suggestive of genome-wide significance. Despite these general considerations, as there are very few GWAS studies that analyze taste preference, some tables show SNPs with *p*-values below 5 × 10^−4^ so as to help other authors compare their results or in undertaking meta-analyses. Although all the participants were white Caucasians, and no ethnicity bias or population stratification was expected, we checked this potential influence by calculating the genomic inflation factor (lambda coefficient: >1 indicates inflation and <1 indicates deflation). Likewise, we carried out a PCA using PLINK [62,63]. This method used genome-wide genotyping data to estimate principal components axes that can be used as covariates in the corresponding association analysis. 20 principal components were computed using PLINK [63] and used the top of these components to adjust the association models.

We used R qqman R library [67] and Haploview (version 4.2) [68] to create Manhattan plots and to calculate LD between the SNPs of interest. A quantile–quantile plot (Q-Q plot) comparing the expected and observed *p*-values [67] was performed in the R-statistical environment. Furthermore, we used FUMA (Functional Mapping and Annotation of Genome-Wide Association Studies) [65] to calculate the lambda statistic for Q-Q plots. We used LocusZoom.js to generate locus-specific graphical displays of the position of the selected SNPs in the GWAS to nearby genes and local recombination hotspots [69], as well as to indicate the LD.

Besides carrying out several SNP-based GWASs, in which associations with individual SNPs are analyzed, we also undertook a gene-based GWAS. This gene-based analysis considers the aggregate effect of multiple SNPs in a single test instead of analyzing each SNP separately [65]. This sets off from the initial association results of the different SNPs with PLINK (summary statistics) and used FUMA, a platform that annotates, prioritizes, visualizes, and interprets GWAS results [65,70,71,72]. This platform uses MAGMA (Multi-marker Analysis of GenoMic Annotation), a fast and flexible tool for gene and gene-set analysis of GWAS genotype data [71]. LD information was retrieved from the 1000 Genomes European panel. Bonferroni correction for the gene-based analysis was set at the gene level significance evidence level of *p* < 2.7 × 10^−6^ (−log_10_ 2.7 × 10^−6^ = 5.568). Likewise, the suggestive level of association for the gene-based analysis was set at *p* < 1 × 10^−4^ [73]. In addition, for the dichotomous sweet taste preference variable, we used the FUMA SNP2GENE tool for extensive functional annotation for all SNPs in genomic areas identified by lead SNPs; and the FUMA GENE2FUNC function for the annotation of genes in biological context [65].

## 3. Results

### 3.1. Participants Characteristics

Table 1 shows the demographic, anthropometric, clinical, biochemical and lifestyle characteristics of the sample studied. All subjects were white Caucasian participants in the PREDIMED Plus-Valencia study. We included 425 subjects that, in addition to the general variables of the study, had taste preference and food preference data available. They consisted of older men and women (mean age 65 ± 4.7 years) with metabolic syndrome. The prevalence of diabetes was 38.4% and the mean BMI was also high, with no differences between men and women (*p* > 0.05 for both).

### 3.2. Preference for Sweet Taste and for Other Tastes

All participants completed the taste preference test, using the 9-point hedonic scale as detailed in methods. On this scale, the higher the score, the higher the preference for each taste. Table 2 shows the means of each taste preference for the whole sample and stratified by men and women. In the whole population, sweet taste was the second most preferred taste, reaching a mean of 7.16 points. The most preferred taste was salty, with a mean of 7.56 points, a score very close to the preference for sweet taste. These tastes were followed at a greater distance by umami (5.95), then sour (4.62 points) and finally bitter (4.31 points), this being the least preferred in this elderly population. In the per sex analysis, no statistically significant differences were observed between men and women in the preference for sweet taste (*p* = 0.245). No differences were also observed in preferences for salty, sour and bitter tastes. However, very significant differences were observed between men and women in their preference for umami (*p* < 0.001), this being more strongly preferred in men than in women. Table 2 also shows the means of taste preferences in type 2 diabetic and non-diabetic subjects. In general, the means of taste preferences are similar in both groups, most differences arising in the preference for sweet taste, which is higher in subjects with type 2 diabetes (*p* = 0.021). This association remained statistically significant in a multivariate analysis after adjustment for sex and age (*p* = 0.020) and after additional adjustment for BMI (*p* = 0.021), as well as after additional adjustment for smoking, physical activity, hours of sleep per night and adherence to the Mediterranean diet score (*p* = 0.033). Likewise, on creating the dichotomous variable of high or low preference for sweet taste (based on the mean), a high preference for sweet taste was associated with a greater likelihood of being diabetic, in a model adjusted for sex and age (OR: 1.59; 95%CI: 1.04–2.41, *p* = 0.031), and for sex, age and BMI (OR: 1.59; 95%CI: 1.04–2.41; *p* = 0.032). Moreover, additional adjustment for smoking, physical activity, hours of sleep per night, and adherence to the Mediterranean diet, did not change the statistical significance level for this association (OR: 1.54; 95%CI: 1.01–2.36; *p* = 0.048). However, on analyzing taste preference depending on the presence or absence of obesity (Table S2), no differences were found between the obese and the non-obese groups, for sweet taste preference (*p* = 0.437) or any other taste.

### 3.3. Perception of Sweet Taste and Other Tastes

Taste perception (Table 3) for different tastes was assessed by tasting in the laboratory and rating the intensity of the perceived taste, as detailed in the methods (the higher the score, the more taste perception).

Taste perception data was only available for 348 of the 425 participants included in this study. As we have already published a previous paper on the association of taste perception with sex and obesity [41], we will not focus on obesity but on type 2 diabetes. Table 3 shows the means for the perceptions of the five taste qualities, as well as for the total taste score according to type 2 diabetes status.

The total taste score, which is the sum of the five taste perceptions, was statistically lower in diabetic subjects (*p* = 0.034 and *p* = 0.019 after multivariable adjustment). On studying specific tastes, statistically significant differences were only observed in bitter taste, its perception being lower in diabetic subjects (1.16 ± 0.11 versus 1.51 ± 0.09; *p =* 0.024), remaining statistically significant after multivariable adjustments (*p* < 0.05). For sweet taste, although perception was slightly lower in diabetic subjects, the differences did not reach statistical significance (*p =* 0.182).

### 3.4. Correlation of Sweet Taste Preference with the Other Tastes

Table 4 presents the Spearman correlation coefficients between sweet taste preference and preference for the other tastes. Although, in general, the correlations were not very high, we observed some statistically significant correlations. Among them, sweet taste preference had a statistically significant direct correlation with salty taste preference (r = 0.119; *p =* 0.014). Similarly, the preference for sweet taste was inversely correlated with the preference for sour taste (r = −0.110; *p =* 0.024). For the other tastes, the highest correlation was observed between the preference for bitter and sour tastes (r = 0.371; *p* < 0.001).

We also analyzed the correlations between taste preference and taste perception. The results are shown in Table S3. Although the preference for sweet taste shows an inverse correlation with sweet taste perception, this association is very small and did not reach the statistical significance (r = −0.056; *p =* 0.297). However, salty taste perception presented an inverse correlation with salty taste preference (r = −0.126; *p =* 0.018). Likewise, a higher umami taste perception was inversely associated with the preference for the umami taste (r = −0.133; *p =* 0.013).

### 3.5. Factor Analysis of the Main Components for Taste Preferences

**Figure 1:**
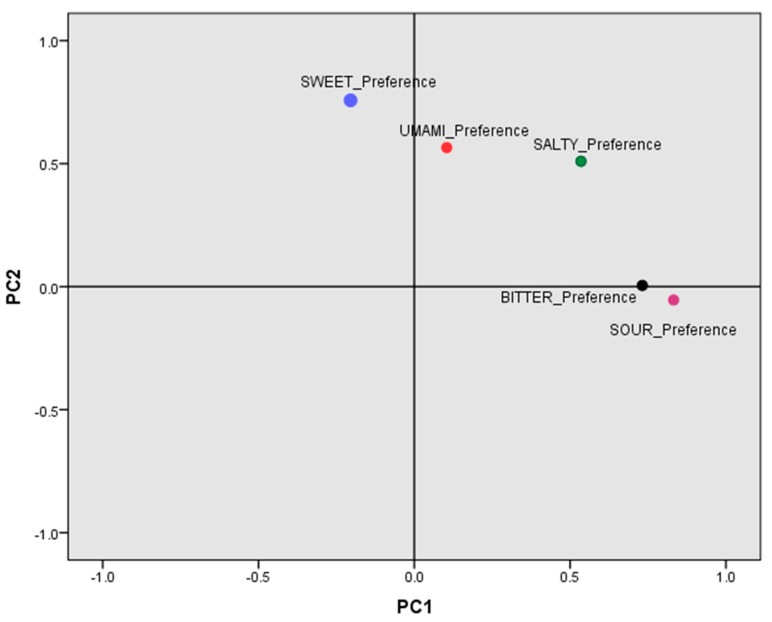
Principal component analysis loading plots for the rotated (varimax rotation) components. Taste preference analysis for sweet, salty, sour, umami and bitter. PC1 (principal component 1), PC2 (principal component 2). Data obtained from a factor analysis including the five taste preference variables (*n* = 425 subjects).

It can be observed that preferences for different tastes are complex and correlated. A factor analysis of the main components, based on the five taste preference variables, was undertaken to better understand the latent structure underlying taste preferences. The KMO measure and the Bartlett test of sphericity (*p* = 2 × 10^−23^) indicated the pertinence of carrying out the factor analysis. Following the Kaiser criterion of extracting factors with eigenvalues > 1, two factors were extracted. The first factor (Factor 1) accounted for 32.1% of the total variance. The second factor (Factor 2) explained 22.3% of the total variance. Having undertaken the varimax transformation to better interpret these latent variables or factors, we observed (Table S4) that Factor 1 presented positive high factor loadings with sour taste (0.832) and bitter taste (0.732) and can be identified as the component that represents sour and bitter tastes. Salty taste also presented a fairly high positive correlation with Factor 1, but lower than the previous ones. High factor loading observed for sour and bitter mainly characterized this factor and was labeled “sour-bitter-preference”. Factor 2 had a high factor loading for sweet preference (0.757) followed by umami (0.565). In the same way as for Factor 1, this Factor 2 also presented high positive correlations with salty taste, but it is not a differential trait, since salty taste also presented high factor loading with Factor 1. Therefore, Factor 2 was labeled “sweet-umami preference”. Figure 1 shows the PCA loading plots for the rotated (varimax rotation) components for this analysis. In this figure, the projection of the taste preference variables can be better appreciated.

### 3.6. Association between Sweet Taste Preference and Liking for Sugary Foods

In the association study between sweet taste preference and liking for sugary foods (“breakfast cereals”, “sweets and ice creams”, “chocolate” and “sugar”), we found strong statistically significant associations for these last three foods in the whole sample, as well as when stratified into diabetic and non-diabetic subjects. Table 5 shows the association between the dichotomous variable of sweet taste preference (high preference vs. low preference) and liking for the four sugary foods included in the questionnaire, both for the whole population and by type 2 diabetes status. Except for the preference for breakfast cereals, for which statistically significant associations were weak, there were highly significant associations (*p <* 0.001) with the other sugary foods. Thus, in the whole population, having a high preference for sweet taste was associated with a higher likelihood of showing a high preference (liking) for “breakfast cereals” (OR: 1.63; *p =* 0.031), “sugar” (OR: 2.35, *p <* 0.001), “chocolates” (OR: 4.47; *p <* 0.001) and “sweets and ice creams” (OR: 14.48; *p <* 0.001), in the unadjusted analysis. These associations (except for the “breakfast cereals”) remained statistically significant after multivariable adjustment (Table 5).

Similar associations between sweet taste preference and liking for sugary foods were found in non-diabetic and in type 2 diabetic subjects. For both groups, a high preference for sweet taste was associated with a very high liking of sweets and ice creams. These foods were followed by a high preference for chocolates, which was relatively higher in type 2 diabetic and in non-diabetic subjects.

### 3.7. Association between Sweet Taste Preference and Sugar-Rich Food Intake

To analyze the association between sweet taste preference and the intake of sugary foods in the diet, we focused on the sugary foods included in the Mediterranean diet score (17-item) (Table S1), as indicated in the Methods section. We selected three items: Item 6 (Sugar-sweetened beverages), Item 9 (Sweets-pastries) and item 13 (Added sugar or non-caloric sweeteners).

Table 6 presents the results of these associations, both in the whole population and depending on type 2 diabetic status. Food intake for each sugary food item was used dichotomously in accordance with the pre-specification of their intake (complying or not with the recommendations of the Mediterranean diet, labeled as low intake). This compliance was as follows: <1 time/week for sweetened beverages; <3 time/week for pastries, and No consumption or use of non-caloric sweeteners (NCS).

In general, we observed that there was a higher intake of sugary foods (mainly sweets-pastries and sugar-sweetened beverages) in those who prefer sweet taste, but the associations did not reach the statistical significance in the whole population. However, statistically significant associations were observed in type 2 diabetic subjects. In these subjects, a high preference for sweet taste was associated with less compliance of the Mediterranean recommendation (50.4% in subjects with high preference for the sweet taste vs. 66.7% in subjects with a low preference for the sweet taste; *p* = 0.013 in the multivariate-adjusted model) for sweetened beverages (less 1 time/week). Thus, according to our estimations, in diabetic subjects with a high preference for sweet taste, the likelihood of having a low consumption of sugar-sweetened beverages (<1 time per week) was decreased (OR: 0.51, *p <* 0.013). Likewise, for pastries, taking into account that the pre-specified consumption for these items in accordance with the adherence to the Mediterranean diet is less than three times per week, diabetic subjects with high preference for the sweet taste had less compliance with the Mediterranean recommendation for this item (38.3% in subjects with high preference for the sweet taste vs. 56.3% in subjects with a low preference for the sweet taste; *p* = 0.039 in the multivariate-adjusted model).

Interestingly, regarding item-13 (“Do you add sugar to the coffee or tea?”), type 2 diabetic subjects, with a high preference for the sweet taste, scored well in this item, mainly due to higher use of non-caloric sweeteners, avoiding sugar.

### 3.8. Exploratory GWASs to Study SNPs and Genes Associated with Sweet Taste Preference

#### 3.8.1. GWASs for Sweet Taste Preference

We used two approaches to analyze the genes and gene variants associated with sweet taste preference. Firstly, we conducted exploratory SNP-based GWASs to identify the SNPs most associated. Secondly, we conducted exploratory gene-based GWASs.

##### SNP-Based GWAS for Sweet Taste Preference

**Figure 2:**
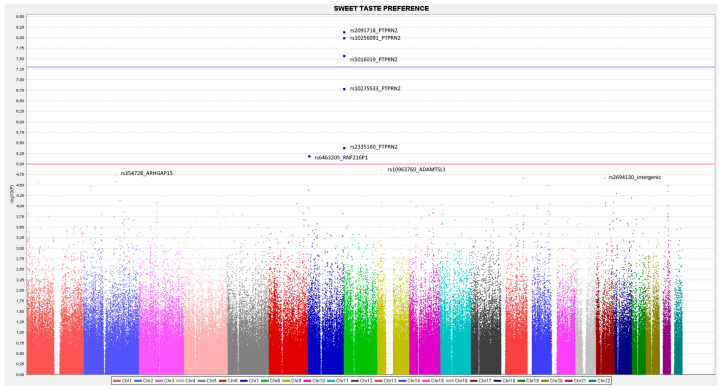
Manhattan plot for the GWAS analysis on sweet taste preference (as a dichotomous variable) in the whole population using PLINK and a single nucleotide polymorphism (SNP)-based GWAS analysis. A genetic additive model and logistic regression were used to estimate *p*-values, and the top-ranked SNPs were annotated. The blue line represents the threshold 1 (−log_10_(5 × 10^−8^)) for the GWAS statistical significance. The red line represents the threshold 2 (−log_10_(1 × 10^−5^)).

First, we analyzed the SNPs at the genome-wide level associated with sweet preference using the dichotomous variable (high or low preference). We used logistic regression analysis to estimate the coefficients, as well as an additive genetic model. Figure 2 presents the corresponding Manhattan plot showing the *p*-value (−log_10_ *p*) of each SNP analyzed in the crude model. Figure S2 shows the corresponding Q-Q plot. The calculated lambda (λ) value was very good (λ = 1.004) suggesting no population stratification bias.

**Figure 3:**
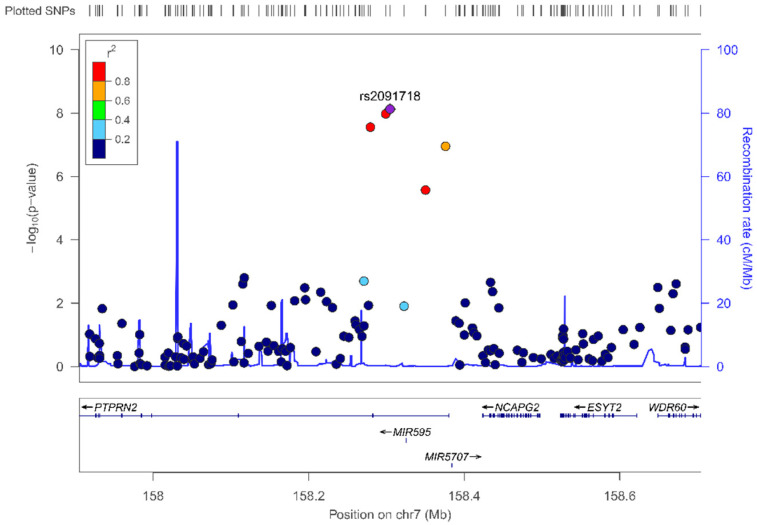
Regional plot for the lead-SNP rs2091718, located in the protein tyrosine phosphatase receptor type N2 (PTPRN2) gene, on chromosome 7, for sweet taste preference (as a dichotomous variable). *p*-values obtained in the logistic regression model adjusted for sex and age.

Three SPNs in the PTPNR2 (Receptor-type tyrosine-protein phosphatase N2) gene (rs2091718, *p* = 7.17 × 10^−9^; rs10256091, *p* = 9.94 × 10^−9^; and rs5016019, *p* = 2.61 × 10^−8^) reached statistical significance at the genome-wide level (*p <* 5 × 10^−8^). The PTPNR2 gene is located at chromosome 7. The MAF for these variants was relatively high (0.245, 0.342 and 0.251, respectively). The top-ranked was the rs2091718- PTPNR2. Figure 3 shows the regional plot for this SNP. Two micro-RNA genes were close to this SNP (MIR595 and MIR5707). In Figure 2, we also considered the suggestive *p*-value for significance (*p* < 1 × 10^−5^), and three SNPs surpassed this level including two other SNPs in the PTPRN2 gene and one SNP in the RNF216P1 (Putative protein RNF216-like) gene (*p <* 5 × 10^−5^).

Table 7 shows more detailed information on the top-ranked SNPs in the GWAS on the sweet taste preference, including the position, the effect (OR), the *p*-value, the corresponding MAF as well as the annotated gene for the SNPs. Estimations in Table 7 have been adjusted for sex and age. After this adjustment, the lead SNP, rs2091718-PTPRN2, remained statistically associated with the sweet taste preference at the genome-wide level, with the minor allele related to a low sweet taste preference (OR: 0.35; *p* = 7.46 × 10^−9^).

Additional adjustment for type 2 diabetes (Table S5), did not change the significance at the GWAS level for the lead SNP rs2091718-PTPRN2 (*p* = 2.28 × 10^−8^), showing a good consistency for the other SNPs. This is the first time that an SNP in the PTPRN2 gene is suggested to be associated with the sweet taste preference. Although the above calculated genomic inflation λ indicated little or no population stratification, we additionally adjusted the regression model for the main principal components (PC) including PC1, PC2, PC3 and PC4, as covariates in the model adjusted for sex and age. Table S6 shows the estimations for Table S5 after this additional adjustment for population stratification. As expected, based on the previous **λ** results, this additional adjustment did not change the statistical significance of the rs2091718-PTPRN2 SNP, which remained at the genome-wide level. Figure S3 shows the Q-Q plot for this GWAS including the PCs as covariates. As the λ value was 1.022 and did not improve the λ value without the PC adjustment, we continued to use the models without this additional adjustment.

To better understand the functionality of these SNPs and related genes, a functional analysis with the FUMA tools [65,72] was undertaken. Figure S4 shows the lead rs2091718-PTPRN2 SNP for the multivariable-adjusted model, its regional plot with the corresponding LDs, the combined annotation dependent depletion (CADD) score for the lead SNP and the region, as well as the Regulome score. This score is a categorical score from *1a* to *7*, showing the likelihood of functionality for the lead SNP and related SNPs.

**Figure 4:**
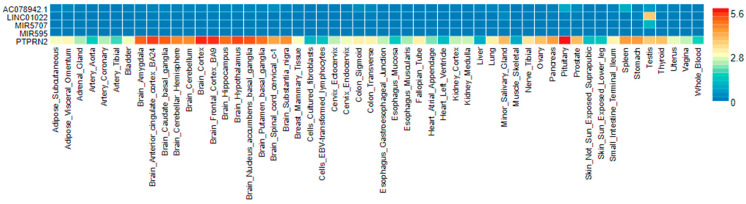
Gene expression heat map based on the data set GTE × V8 (54 tissue types), showing the average expression per label for the expression of the PTPRN2 gene and the regional overlapping genes MIR5707, MIR595, AC078942.1 and LINC01022.

Based on the genes nearby and the lead SNP in the regional plot (Figure S4) we obtained with FUMA a gene expression heatmap using the GTEx V8 (54 tissue-types) dataset and showing the average expression per label (log_2_ transformed) (Figure 4). This functional analysis revealed interesting findings for the PTPRN2 gene. This gene is widely expressed in brain tissues, mainly in the brain-cortex. In addition, it is expressed in the minor salivary gland and the stomach tissues.

##### Gene-Based GWAS for Sweet Taste Preference

**Figure 5:**
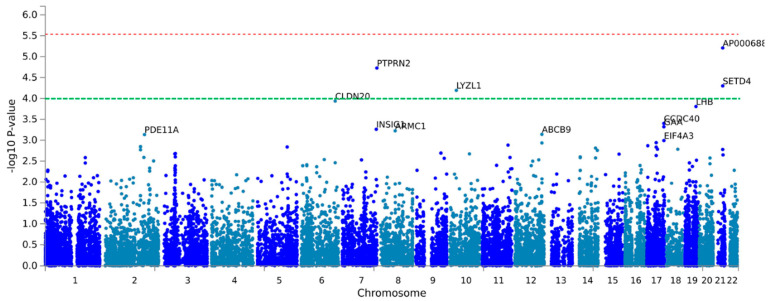
Manhattan plot for the GWAS analysis for sweet taste preference (as a dichotomous variable) in the whole population using a gene-based approach (adjusted for sex and age). We used MAGMA for computations in the FUMA platform. The top-ranked genes were annotated. The red line at 5.56 represents the threshold 1 (−log_10_(2.7 × 10^−6^)) for the gene-set analysis considering the strict Bonferroni correction. Likewise, the green line at 4.00 represents the threshold 2 (−log_10_(1 × 10^−4^)).

Figure 5 shows the Manhattan plot for the exploratory gene-based GWAS for sweet taste preference (dichotomous variable) in the model adjusted for sex and age. In this approach, SNPs in each gene were considered at the same time using MAGMA [71]. Figure S5 shows the Q-Q plot for this gene-based GWAS.

Here, the *p*-values for statistical significance at the genome-wide level are different from the SNP-based GWAS: being *p* < 2.7 × 10^−6^ and *p* < 1 × 10^−4^ for gene-based GWAS significance and for the suggestive level, respectively. In Figure 5, the red line indicated the −log_10_(*p*-value) at the gene-based GWAS statistical significance, and the green line, the suggestive level of association. These results at the gene-based level are consistent with our SNP-based GWASs for the PTPRN2 gene. This gene appears as the second top-ranked gene at *p =* 1.88 × 10^−5^ (suggestive level of significance). The first top-ranked gene (also at the suggestive level of significance) was the AP000688.1 (*p* = 6.23 × 10^−6^) gene at chromosome 21. This gene corresponds to a long-non-coding RNA and has a relatively small size in comparison with the PTPRN2 gene. Other genes reaching the suggestive gene-based GWAS level were: SETD4 (SET domain containing 4) and LYZL2 (lysozyme like 2). SNPs in these genes were also among the top-ranked in the SNP-based GWAS (Table S5).

Table S7 shows the detailed information of the top-ranked genes displayed in the gene-based Manhattan plot, indicating the location, size, and *p*-value. The PTPRN2 gene appears as the second top-ranked gene at *p =* 1.88 × 10^−5^, in the model adjusted for sex and age. Additional adjustment of the model for type 2 diabetes did not change the level of significance for the PTPRN2 gene (*p* = 3.94 × 10^−5^).

Functional analysis, consisting of the gene expression heat map based on the data set GTE × v8 (54 tissue types) [65], for the PTPRN2 gene and other top-ranked genes (Figure S5), showed that the PTPRN2, widely expressed in brain tissues, mainly in the brain cortex and brain hypothalamus, has a similar a gene expression profile than the SETD4 gene, and both differed from the LYZL2 gene.

#### 3.8.2. GWAS for Factor 2

Additionally, we undertook an exploratory SNP-based GWAS on the factorial scores (as a continuous variable) for Factor 2, to analyze the gene variants that are associated with sweet taste preference, but also considering preferences for other tastes. Factor 2 has been identified as having a high preference for sweet taste, followed by umami, and a low preference for bitter and sour tastes. Table S8 shows the results of this GWAS in a model adjusted for sex and age. The lead SNP (rs2694130) has a small MAF and did not reach the *p*-value of GWAS significance. Most importantly, several SNPs in the PTPRN2 gene were among the top-ranked SNPs (the rs5016019-PTPRN2 with *p* = 9.16 × 10^−6^; rs2091718-PTPRN2 with *p* = 2.01 × 10^−5^), suggesting additional relevance of this gene on the sweet taste preference.

### 3.9. Association of rs2091718-PTPRN2 SNP with Sweet Taste Preference Variables and Other Related Variables

Once the rs2091718-PTPRN2 polymorphism had been identified as the lead SNP in the SNP-based GWAS for the dichotomous variable for the sweet taste preference in the logistic regression model, we analyzed this association in depth with other sweet taste preference variables and related traits. For this SNP, the genotype frequencies (A > G) were: 58.8% AA, 36.0% AG and 5.1% GG; and was in a high Hardy-Weinberg equilibrium in this sample (*p* = 0.625).

**Figure 6:**
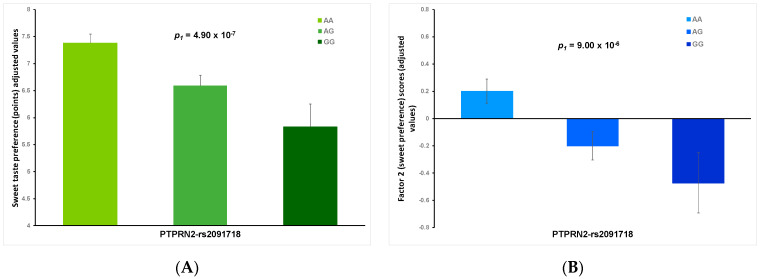
Adjusted means for sweet taste preference (Panel (**A**): using the sweet taste preference variable as continuous; and Panel (**B**): using the factor 2 scores) depending on the lead SNP rs2091718, located in the protein tyrosine phosphatase receptor type N2 (PTPRN2) gene in the whole population. Adjusted means and *p*-values (*p*_1_) have been obtained in a general linear regression model adjusted for sex, age, type 2 diabetes, BMI, smoking, physical activity, hours of sleep per night and adherence to Mediterranean diet. Minor allele frequency (MAF) is 0.24 for the G allele in this population. Genotype prevalence: AA (58.1%); AG (38.8%); and GG (5.1%).

Figure 6 shows the adjusted means for sweet taste preference as a continuous variable (panel A) and the adjusted factorial scores for Factor 2 (panel B), depending on the rs2091718-PTPRN2 SNP in a model adjusted for sex, age, type 2 diabetes, BMI, smoking, physical activity, hours of sleep and adherence to the Mediterranean diet. For both variables, high statistically significant associations were found, adding consistency to the GWASs findings.

Furthermore, when we examined the association between the rs2091718-PTPRN2 SNP and sweet taste preference, according to diabetes status, we obtained statistically significant results (*p* < 0.01) in both non-diabetic and type 2 diabetic subjects (not shown).

Lastly, we analyzed if the rs2091718-PTPRN2 SNP was associated with type 2 diabetes prevalence in this population. Using a genetic additive model, the minor G-allele was associated with a decreased likelihood of being a subject with type 2 diabetes, both in the crude model (OR: 0.70; 95%CI: 0.50–0.97, *p* = 0.034), and even after multivariable adjustment for sex, age, BMI, smoking, physical activity, hours of sleep and adherence to the Mediterranean diet (OR: 0.69; 95%CI: 0.49–0.97, *p* = 0.032).

## 4. Discussion

This study carried out in elderly Mediterranean subjects with metabolic syndrome contributes new insights into sweet taste preference and related variables, so adding more clues to the emerging field of taste perception, taste preferences and dietary intake for personalized nutrition [4,5]. We found that the sweet taste preference in these subjects is very high, second only to salty taste. In addition, we have observed a strong association between sweet taste preference and the reported preference for sugary foods in this population. However, we did not detect a significant difference in sweetness perception and sweet taste preference. Previous studies analyzing this association have reported diverse results depending on the population analyzed and the methodology used [18,19,24,25]. Contextualizing the age of the population is important when carrying out studies on taste perception or taste preferences, as there may be key differences in the results obtained and, in the conclusions drawn. Most studies on taste perception and taste preferences have been carried out in children or in the young or middle-aged population [7,8,9,18,20,23,25,29], so it is valuable to provide more information on the taste preference in the elderly population, just as we do in the present work. Studies indicate that the preference for different tastes varies according to age and that the preference for sweet taste is higher in children [25,31]. It is also known that there are age differences in the ability to perceive tastes, and thus, in a previous study that was undertaken on a Mediterranean population with subjects between 18 and 80 years old [61], we were able to show that in general, there is a decrease in taste acuity with age, which is more noted for some tastes than others, but that, in general, the “total taste score” that reflects the contribution of all five tastes decreased significantly with age.

In addition to age, the characteristics of the population in terms of the existence or non-existence of pathologies are also important [32]. Hence, one important characteristic may be the presence of subjects with type 2 diabetes in the population studied. Although it is generally believed that people with diabetes may differ from non-diabetic subjects in their ability to perceive tastes, or in their preferences for certain tastes, the studies carried out on this aspect have been scarce and the conclusions are diverse [48,49,50,51,52,53,74]. Our study found that subjects with type 2 diabetes have a slightly lower taste perception for all tastes combined in the computed “total taste score”, with bitter taste being the only taste quality showing statistically significant differences. Impairment of taste sensation in type 2 diabetic subjects [51] has previously been reported and there is some agreement [48,51,53] in recent studies on general taste perception declining, but differences among studies persist on the most affected taste quality in diabetic subjects [48,49,50,51,52,53,74].

Moreover, in the present study, we detected a slightly higher statistically significant preference for sweet taste in subjects with type 2 diabetes. Although patients with diabetes are generally thought to prefer sweet tastes, little data are available [74]. Pugnaloni et al. [53] hypothesized that a broad alteration of taste function can lead patients with type 2 diabetes to search for sugary foods, so increasing the dietary risk. In agreement with this hypothesis, when we analyzed food intake in the present study analysis, focusing on the sugary foods included in our Mediterranean diet scale [56], we found some significant associations between a higher preference for the sweet taste and a higher intake of sugar-sweetened beverages and sweets-pastries in type 2 diabetic subjects. However, added sugar intake tended to be lower in type 2 diabetic subjects with a high preference for sweet taste (mainly due to the use of low-calorie sweeteners instead of added sugar). No associations between sweet taste preference and the foods analyzed were detected in non-diabetic participants. Therefore, type 2 diabetes should be a trait to consider when comparing results of sweet taste perception, sweet taste preferences and sugary food intakes between different populations and for subsequent personalized nutrition. Several studies have reported an increased consumption of sugary foods in subjects with a higher preference for sweets [19,21,23,24,25], and subsequently, a diet high in sugary foods has been related to obesity and a higher risk of type 2 diabetes [1,2,3]. However, this association is complex and highly dependent on the context [9,23,32,74]. Thus, more standardization and focus on the population characteristics is needed in further studies.

Although genetic factors are considered relevant for sweet taste preferences [22,30,37], the main genes and genetic variants associated with sweet taste preference remain mostly underexplored at the GWAS level. Very few cohorts conducting GWASs have measured the sweet taste preference variable [40,45]. Even in studies that analyze the association between sweet taste preference and different phenotypes, great heterogeneity is found in the measuring methods [18,23,24,32,40,44,45,46]. In the present work, the measurement of sweet taste preference was undertaken by a 9-point hedonic scale with 9 increasing scores of preference in the context of the measure of the other tastes, whereas other GWASs have used an internet-based question, consisting of a five-point scale of preference [45], only asking for sweet preference or other methods to quantify this preference [40]. Moreover, some GWAS have not even specifically asked about the preference for sweet taste [40,44,46], but have assumed that the intake of sweet food indicates the preference for that taste. Thus, several sugary food proxies for the sweet taste preference, have been analyzed at the GWAS level [40,44,46]. This poses a great disadvantage when it comes to making comparisons as different constructs are being measured and the results can be very heterogeneous. It would, therefore, be necessary to standardize measurements in this field of research. Therefore, due to this heterogeneity, consistency in the top-ranked SNPs identified for sweet taste preference is very low.

Moreover, the geographic origin of the population and ancestry differences may have a relevant influence when comparing results. Taking into account that very few GWASs have been carried out in the Southern European Mediterranean populations and that this population may differ in several ancestry characteristics from the Northern European populations [47], specific studies are needed in the Mediterranean population for better precision medicine. Although our sample size is small, we undertook an exploratory GWAS study in this population to obtain preliminary results and some clues on the main SNPs and genes related to sweet taste preference in elderly Mediterranean subjects with metabolic syndrome. In a previous GWAS carried out in this population we were able to detect a high statistically significant association between bitter taste perception and SNPs in the TAS2R38 gene [41]. Although the association between sweet taste preference and genetic factors is not expected to be as strong as the relationship with bitter taste, we obtained a statistically significant result at the genome-wide level. We identified several SNPs in the PTPRN2 gene (located at chromosome 7), significantly associated with sweet taste preference. The hit SNP was the rs2091718 SNP, remaining statistically significant at the genome-wide level after adjustment for several factors, including type 2 diabetes (*p* = 2.28 × 10^−8^). To minimize the fact that the finding might be due to chance, we fitted several regression models (including a GWAS on the combined Factor 2 linked to sweet the taste preference in the context of other tastes) and controlled for population stratification, obtaining similar results. Moreover, we conducted an additional gene-based GWAS for sweet taste preference and the PTPRN2 gene was the second top-ranked at the suggestive level of significance in this analysis. This is the first time that this gene has been reported to be associated with sweet taste preference and more studies in this and other populations are needed for replication or to extend findings. Overall, the PTPRN2 gene is a promising candidate gene. This gene encodes a major islet autoantigen in type-1 diabetes and plays an important role in insulin secretion in response to glucose stimuli [75,76] and β-cell transcription and proliferation [77].

In animal models the deletion of the PTPRN2 gene was associated with a reduction in insulin secretion as well as with a decrease of the neurotransmitters serotonin, norepinephrine, and dopamine, influencing changes in learning and behavior in mice [78]. There are previous studies in humans, relating this gene with cocaine addiction and depression [79], as well as with bipolar disorders [80] and with developmental disorders in children [81]. Moreover, it has been published that one of the genes in the human genome that contains more “variable number tandem repeats” (VNTRs), a type of structural regulatory variant, is the PTPRN2 gene [82]. The VNTRs variants are related to many diseases, mainly affecting the nervous system and several obesity phenotypes [83,84,85]. Other characteristics for the functionality of the VNTRs on pathogenicity that fulfills the PTPRN2, is the subtelomeric location (the PTPRN2 is positioned within the subtelomeres of chromosomes 7 and 8), as well as a predominant gene expression in the brain [82], as we have observed in the functional gene expression analysis that we have carried out in this study. These functional characteristics of the PTPRN2 gene are in agreement with our novel finding relating this gene to the preference for sweet taste. Additional work is needed to better characterize the molecular mechanisms involved.

Additionally, considerable new information has accumulated over the last few years on the role of the PTPRN2 in the epigenetic regulation of metabolic diseases, neurodegeneration, and cancers. Differential methylation of the PTPRN2 gene and higher risk of obesity in children [86], type 2 diabetes [87], cognitive decline [88], future cardiovascular risk [89], and cancer [90,91], have been reported in some studies. In the present work, we did not analyze methylation and we do not know if the association between the top-ranked PTPRN2 SNPs and sweet taste preference is modulated by this epigenetic mechanism.

Interestingly in the present study, the lead SNP rs2091718 in the PTPRN2 gene has been significantly associated with type 2 diabetes prevalence. Carriers of the minor G-allele for this variant presented a lower likelihood of diabetes. Likewise, G-carriers had a lower sweet taste preference. Although several animal studies and methylation studies in humans have reported associations between the PTPRN2 gene and type2 diabetes [75,76,87,92,93], genetic associations at the SNP level are very scarce. In absence of mechanistic investigation, it is difficult to know if the association between the rs2091718-PTPRN2 polymorphism and type 2 diabetes is a cause or a consequence. However, when we examined the association between the rs2091718-PTPRN2 SNP and sweet taste preference depending on the diabetes status, we obtained statistically significant associations in both non-diabetic and type 2 diabetic subjects. Another interesting work to mention here is the study carried out in participants in the Mars-500 mission to investigate Psycho-Epigenome-Metabolism changes during adaptation to long-term isolation [94]. They analyzed longitudinal DNA methylation patterns to explore glucose- and mood-state-synchronized DNA methylation sites under extreme isolation conditions. Interestingly, the PTPRN2 gene was consistently found to cover DNA methylation sites synchronously changing with glucose and mood-state. This result supports our finding, suggesting the PTPRN2 gene as a novel relevant gene in determining preference for sweet taste, and also potentially associated with fasting glucose. Curiously, in a recent study, the PTPRN2 gene has been identified as one of the most relevant genes related to the genomic architecture of the feeding efficiency in chickens [95].

With regard to other genetic determinants of sweet taste preferences, initial studies analyzed associations between several SNPs in candidate genes, such as TAS1R1, TAS1R2, TAS1R3, GRM1, SLC2A2, DRD2, OPRM1, SCNN1A, SCNN1B, SCNN1G, SCNN1D, TRPV1, and GNAT3, among others), with mixed results [32,33,34,35,36,37,42,43,44]. In our GWASs we did not detect any of these genes as top-ranked, indicating a small association, if any. A detailed analysis focusing on these variants will be carried out to further evaluate such potential associations and the potential modulation by type 2 diabetes. Understanding individual preferences for sweet taste and the factors related to food intake is of growing interest and importance for personalized nutrition and precision medicine in diverse populations [96,97].

## 5. Conclusions

In conclusion, our results indicate that sweet taste preference in elderly subjects from this Mediterranean population, is still high, second only to salty taste, and suggest that the preference for the sweet taste is slightly higher in type 2 diabetic compared to non-diabetic subjects. In terms of taste perception, the total taste score was slightly lower and statistically significant in type 2 diabetic subjects, this difference being more evident for bitter taste perception. Individual preferences for the five tastes were correlated, and a latent structure can be extracted by factor analysis. The sweet taste preference was highly associated with sugary food preferences. In our exploratory GWASs analyses (SNP-based and gene-based), we detected a promising candidate gene, the PTPRN2, associated with sweet taste preference. This is the first time that such a suggestive association has been reported, and more studies in other populations are required to confirm these findings. The PTPRN2 gene is highly expressed in brain tissues, our results contributing to the idea that genes, apart from those within the peripheral sweet receptor pathways, may also be involved in determining sweet taste preferences. Thus, our results may be useful as a starting point for other investigations.

## Figures and Tables

**Table 1:** Demographic, clinical, and genetic characteristics of the study population according to sex.

Characteristics	Total (*n* = 425)	Men (*n* = 183)	Women (*n* = 242)	*p*
Age (years)	65.2 ± 4.7	64.0 ± 5.3	66.1 ± 4.1	<0.001
Weight (Kg)	83.9 ± 13.7	92.5 ± 13.2	77.3 ± 9.8	<0.001
BMI (Kg/m^2^)	32.2 ± 3.6	32.2 ± 3.4	32.2 ± 3.7	0.961
Waist circumference (cm)	105.6 ± 10.1	111.1 ± 8.7	101.4 ± 9.0	<0.001
SBP (mm Hg)	141.3 ± 18.1	143.8 ± 18.6	139.5 ± 17.6	0.015
DBP (mm Hg)	80.9 ± 9.9	82.7 ± 10.4	79.5 ± 9.4	0.001
Total cholesterol (mg/dL)	196.8 ± 37.8	188.3 ± 39.0	203.3 ± 35.6	<0.001
LDL-C (mg/dL)	130.9 ± 33.2	131.5 ± 33.7	130.7 ± 32.8	0.681
HDL-C (mg/dL)	59.9 ± 14.3	52.4 ± 11.3	64.8 ± 13.9	<0.001
Triglycerides (mg/dL)	103.3 ± 58.3	117.7 ± 69.9	93.9 ± 47.1	<0.001
Fasting glucose (mg/dL)	92.1 ± 16.9	94.0 ± 17.9	90.8 ± 16.2	0.001
Physical Activity (MET.min/wk)	1679 ± 1526	1947 ± 1797	1476 ± 1250	0.002
Hours of sleep per night (h/day) ^1^	6.8 ± 1.1	6.9 ± 1.0	6.7 ± 1.1	0.014
Adherence to MedDiet ^2^	8.0 ± 2.8	7.9 ± 2.8	8.1 ± 2.7	0.404
Type 2 diabetes: *n*, %	163 (38.4)	71 (38.8)	92 (38.0)	0.870
Obesity: *n*, %	239 (56.2)	110 (60.1)	129 (53.3)	0.422
Current smokers: *n*, %	48 (11.3)	30 (16.4)	18 (7.4)	<0.001

Values are mean ± SD for continuous variables and number (%) for categorical variables. BMI: body mass index; SBP: systolic blood pressure; DBP: diastolic blood pressure; LDL-C: high-density lipoprotein cholesterol; HDL-C: low-density lipoprotein cholesterol; MET: Metabolic Equivalent. 1 MET is equivalent to kcal·kg^−1^·h^−1^, the oxygen cost of sitting still measured as 3.5 mL/kg/min; *p*: *p*-value for the comparisons (means or %) between men and women. Student’s *t* test was used to compare means and Chi squared tests were used to compare categories. ^1^: Hours of sleep per night on weekdays. ^2^: Quantitative 17-item questionnaire for adherence to Mediterranean diet.

**Table 2:** Taste preferences (sweet, salty, sour, umami and bitter) in the whole population and stratified by sex and type 2 diabetes.

Taste Preference	Total (*n* = 425)	Sex			Diabetes		
		Men (*n* = 183)	Women (*n* = 242)	*p* ^1^	No (*n* = 262)	Yes (*n* = 163)	*p* ^2^
Sweet	7.16 ± 0.09	7.03 ± 0.14	7.25 ± 0.12	0.245	6.99 ± 0.12	7.42 ± 0.14	0.021
Salty	7.56 ± 0.08	7.45 ± 0.13	7.65 ± 0.10	0.196	7.54 ± 0.10	7.60 ± 0.12	0.690
Sour	4.62 ± 0.10	4.71 ± 0.16	4.55 ± 0.14	0.445	4.66 ± 0.13	4.55 ± 0.17	0.614
Umami	5.95 ± 0.09	6.44 ± 0.13	5.58 ± 0.12	<0.001	6.00 ± 0.11	5.87 ± 0.14	0.482
Bitter	4.31 ± 0.10	4.47 ± 0.16	4.19 ± 0.14	0.184	4.33 ± 0.13	4.28 ± 0.17	0.830

Values are mean ± SE; Taste preferences scores have been obtained by a 9-point hedonic scale for each taste. *p* ^1^: *p*-values for sex differences (Student’s *t*-tests). *p* ^2^: *p*-values for diabetes status differences (Student’s *t*-tests).

**Table 3:** Taste perception (sweet, salty, sour, umami and bitter) in the whole population and stratified by type 2 diabetes.

Taste (Tastant) ^1^	Total (*n* = 348)	Diabetes				
		No (*n* = 220)	Yes (*n* = 128)	*p* ^2^	*p* ^3^	*p* ^4^
Sweet (Sucrose) (400 mM)	2.27 ± 0.07	2.34 ± 0.08	2.16 ± 0.11	0.182	0.173	0.171
Salty (NaCl) (200 mM)	2.58 ± 0.07	2.65 ± 0.09	2.44 ± 0.13	0.182	0.168	0.118
Sour (Citric acid) (34 mM)	2.51 ± 0.07	2.58 ± 0.08	2.39 ± 0.12	0.197	0.191	0.132
Umami ((MPG) (200 mM)	1.99 ± 0.07	1.99 ± 0.09	1.99 ± 0.12	0.969	0.979	0.898
Bitter (PTC) (5.6 mM)	1.38 ± 0.07	1.51 ± 0.09	1.16 ± 0.11	0.024	0.023	0.011
Total taste score ^5^	10.71 ± 0.23	11.10 ± 0.29	10.05 ± 0.39	0.034	0.026	0.019

Values are mean ± SE; *p*-values for diabetes status difference were obtained by Student’s *t*-test. PTC: phenylthiocarbamide. MPG: L-glutamic acid monopotassium salt monohydrate. ^1^: Five representative tastants for the five tastes (PTC for bitter, sucrose for sweet, NaCl for salty, citric acid for sour and MPG for umami) were tested (Concentration V). ^2^: *p*-value was obtained in an unadjusted general linear model. ^3^: *p*-value was obtained in a general linear model adjusted for sex, age and BMI. ^4^: *p*-value was obtained in a general linear model adjusted for sex, age, BMI, physical activity, adherence to the Mediterranean diet (17 item score), sleeping hours and tobacco smoking. ^5^: Total taste score: the sum of the scores for the five tastes, at the higher concentration used (concentration V).

**Table 4:** Association between the preference for different tastes.

Taste ^1^		Sweet	Salty	Sour	Umami	Bitter
Salty	r	0.119	1			
	*p*	*0.014*				
Sour	r	−0.110	0.296	1		
	*p*	*0.024*	*<0.001*			
Umami	r	0.017	0.122	0.041	1	
	*p*	*0.730*	*0.012*	*0.402*		
Bitter	r	0.032	0.196	0.371	0.018	1
	*p*	*0.508*	*<0.001*	*<0.001*	*0.714*	

^1^: Taste preferences scores have been obtained by a 9-point hedonic scale for each taste in the whole population (*n* = 425). r: the Spearman correlation coefficient. *p*: the *p*-value for the Spearman correlation coefficient (r).

**Table 5:** Association between sweet taste preference (low/high) and liking for sugary foods (low/high) in the whole population and by type 2 diabetes status.

	Sweet Taste Preference ^1^					
	Whole Population					
**Sugary Foods ^2^**		**Taste Preference ^1^ (%)**		**OR ^3^ and 95% CI**	***p* ^4^**	***p* ^5^**
	**Food** **Liking ^2^**	**Low**	**High**			
Breakfast cereals	High	24.3%	34.4%	1.63 (1.04–2.55)	0.031	0.055
Sweets-pastries and ice creams	High	64.5%	96.3%	14.48 (7.10–29.58)	<0.001	<0.001
Chocolates	High	76.3%	93.8%	4.67 (2.52–8.66)	<0.001	<0.001
Sugar	High	50.7%	70.7%	2.35 (1.55–3.55)	<0.001	<0.001
	**Non Diabetic Subjects**					
	**Food Liking ^2^**	**%**		**OR ^3^ and 95% CI**	***p* ^4^**	***p* ^5^**
		**Low**	**High**			
Breakfast cereals	High	30.8%	43.0%	1.70 (1.01–2.67)	0.045	0.074
Sweets-pastries and ice creams	High	63.5%	96.8%	17.62 (6.64–46.76)	<0.001	<0.001
Chocolates	High	74.0%	92.4%	4.27 (2.05–8.89)	<0.001	<0.001
Sugar	High	51.9%	71.5%	2.32 (1.39–3.90)	0.001	0.003
	**Type 2 Diabetic Subjects**					
	**Food Liking ^2^**	**%**		**OR ^3^ and 95% CI**	***p* ^4^**	***p* ^5^**
		**Low**	**High**			
Breakfast cereals	High	10.4%	22.6%	2.51 (0.90–6.99)	0.071	0.108
Sweets-pastries and ice creams	High	66.7%	95.7%	11.00 (3.74–32.35)	<0.001	<0.001
Chocolates	High	81.3%	95.7%	5.08 (1.60–16.80)	0.003	0.015
Sugar	High	49.9%	69.9%	2.48 (1.24–4.96)	0.009	0.015

^1^: Sweet taste preference was measured in the whole population (*n* = 425) including non-diabetic (*n* = 262) and diabetic subjects (*n* = 163). A dichotomic variable for sweet taste preference was derived consisting of two categories, low preference and high preference, depending on the mean obtained in the 9-point hedonic scale for sweet taste. ^2^: Low and high liking for sugary foods was obtained from a 4-point Likert scale in the food preference questionnaire. Here, a dichotomic (low and high) variable was derived by collapsing the two corresponding categories (1 and 2 vs. 3 and 4). ^3^: OR: Odds ratio; CI: Confidence interval. OR were calculated expressing the probability of a high liking for the corresponding foods depending on a high preference for the sweet taste. ^4^: *p*-value obtained in the unadjusted model. ^5^: *p*-value obtained in the multivariable logistic model adjusted for sex, age, BMI, physical activity, adherence to the Mediterranean diet, sleeping hours and tobacco smoking.

**Table 6:** Association between sweet taste preference (low and high) and intake of sugary foods included in the Mediterranean diet scale. Analysis in the whole population and by type 2 diabetes status.

	Sweet Taste Preference ^1^					
	Whole Population					
**Intake of Sugary Foods in The Mediterranean Diet Scale ^2^**	**Low Intake ^3^ (Med Diet Adherence)**	**Preference ^1^**		**OR ^4^ and 95% CI**	***p* ^5^**	***p* ^6^**
		**Low**	**High**			
Sugary beverages (I-6)	<1/week	58.6%	56.8%	0.93 (0.62–1.39)	0.723	0.476
Pastries (I-9)	<3/week	47.4%	42.1%	0.81 (0.54–1.21)	0.297	0.385
Added sugar (I-13)	No or NCS	63.2%	71.8%	1.48 (0.97–2.26)	0.066	0.105
	**Non Diabetic Subjects**					
	**Low Intake ^3^ (Med Diet Adherence)**	**Preference ^1^**		**OR ^4^ and 95% CI**	***p* ^5^**	***p* ^6^**
		**Low**	**High**			
Sugary beverages (I-6)	<1/week	54.8%	61.4%	1.31 (0.79–2.17)	0.289	0.299
Pastries (I-9)	<3/week	43.3%	44.9%	1.07 (0.65–1.76)	0.790	0.418
Added sugar (I-13)	No or NCS	56.7%	59.5%	1.12 (0.68–1.85)	0.657	0.694
	**Type 2 Diabetic Subjects**					
	**Low Intake ^3^ (Med Diet Adherence)**	**Preference ^1^**		**OR ^4^ and 95% CI**	***p* ^5^**	***p* ^6^**
		**Low**	**High**			
Sugary beverages (I-6)	<1/week	66.7%	50.4%	0.51 (0.25–1.03)	0.057	**0.013**
Pastries (I-9)	<3/week	56.3%	38.3%	0.48 (0.24–0.95)	**0.035**	**0.039**
Added sugar (I-13)	No or NCS	77.1%	88.7%	2.33 (0.96–5.66)	0.057	0.125

^1^: Sweet taste preference was measured in the whole population (*n* = 425) including non-diabetic (*n* = 262) and diabetic subjects (*n* = 163). A dichotomic variable for sweet taste preference was derived consisting of two categories, low and high preference, depending on the mean obtained in the 9-point hedonic scale for sweet taste. In this analysis, a dichotomic variable for sweet taste preference was derived consisting of two categories (low preference for sweet taste and high preference), depending on the mean. ^2^: Intake of the sugary foods included in the 17-item Mediterranean diet scale was considered for analyzing consumption. These items are as follows: sugary beverages (Item-6), sweets-pastries (Item-9), and added sugar (Item-13) (see Table S1 for details). The corresponding food intake was scored into two categories depending on the criteria for adherence to the Mediterranean diet. ^3^: Low intake of these sugary foods was considered for the following frequencies: <1 time/week for sweetened beverages; <3 time/week for pastries, and No consumption or use of non-caloric sweeteners (NCS). ^4^: OR: Odds ratio; CI: Confidence interval. OR were calculated expressing the probability of having an intake according to the Mediterranean diet adherence (labeled as low intake) for the corresponding sugary food depending on a high preference for the sweet taste. ^5^: *p*-value obtained in the unadjusted model. ^6^: *p*-value obtained in the multivariable logistic model adjusted for sex, age, BMI, physical activity, total adherence to the Mediterranean diet, sleeping hours and tobacco smoking.

**Table 7:** Top-ranked SNPs in the GWAS for sweet taste preference (categorical variable) in the whole population. Model adjusted for sex and age.

Chr	SNP	BP	OR	P	Alleles	MAF	Strand	Gene
7	rs2091718	158304646	0.347	7.460 × 10^−9^	G	0.245	−	PTPRN2
7	rs10256091	158299094	0.352	1.054 × 10^−8^	G	0.342	+	PTPRN2
7	rs5016019	158279412	0.364	2.773 × 10^−8^	G	0.251	+	PTPRN2
7	rs10275533	158376086	0.399	1.111 × 10^−7^	A	0.281	+	PTPRN2
7	rs2335160	158350293	0.445	2.683 × 10^−6^	G	0.260	−	PTPRN2
7	rs6463205	5022223	4.078	3.347 × 10^−6^	T	0.105	+	RNF216P1
9	rs10963760	18787794	0.480	9.748 × 10^−6^	G	0.259	+	ADAMTSL1
2	rs354728	143944775	0.513	1.461 × 10^−5^	T	0.206	−	ARHGAP15
17	rs2694130	38747318	0.251	1.560 × 10^−5^	T	0.046	+	__
13	rs971604	97068019	3.888	1.710 × 10^−5^	T	0.186	+	HS6ST3
2	rs10178148	144000004	0.505	2.759 × 10^−5^	G	0.144	+	ARHGAP15
1	rs319978	49067379	0.430	2.796 × 10^−5^	T	0.168	−	AGBL4
17	rs8082554	78039867	0.510	3.642 × 10^−5^	T	0.181	+	CCDC40
7	rs12667108	5133936	0.419	3.661 × 10^−5^	T	0.144	+	__
9	rs10811261	19882156	2.258	4.086 × 10^−5^	G	0.384	+	SLC24A2
11	rs3763872	9593427	0.531	4.329 × 10^−5^	T	0.406	−	WEE1
21	rs2835220	37367098	1.954	4.769 × 10^−5^	C	0.283	+	LOC101928269
14	rs1286470	91059658	0.479	4.955 × 10^−5^	C	0.213	−	TTC7B
2	rs10187143	34022970	0.500	5.180 × 10^−5^	A	0.326	+	LINC01317

Chr: Chromosome. SNP: Single nucleotide polymorphism. BP: Base position in the chromosome (Homo Sapiens GRCh37.p13 genome build used in Illumina HumanOmniExpress-24 BeadChip). OR: odds ratio, indicates the effect for the minor allele on taste preference. P: *p*-value obtained in the multivariable logistic regression model adjusted for sex and age for each SNP using a genetic additive model. MAF: minor allele frequency.

## Data Availability

Neither the participants’ consent forms nor ethics approval included permission for open access. However, we follow a controlled data-sharing collaboration model, and data for collaborations will be available upon request pending application and approval. Investigators who are interested in this study can contact the corresponding author.

## Supplementary Materials

The following are available online at https://www.mdpi.com/article/10.3390/biomedicines10010079/s1, Figure S1: Graphic diagram representing the study design, including the sequences of experiments and statistical analysis carried out; Figure S2: Q-Q plot for the SNP-based GWAS on sweet taste preference (dichotomous variable) in the whole population; Figure S3: Q-Q plot for the SNP-based GWAS on sweet taste preference (dichotomous variable) in the whole population (adjusted for sex, age, type 2 diabetes and genetic PC1, PC2, PC3, PC4); Figure S4: Regional plot for the lead SNP PTPRN2-rs2091718, on chromosome 7; Figure S5: Q-Q plot for the gene-based GWAS on the sweet taste preference (adjusted for sex and age); Figure S6: Gene expression heat map based on the data set GTE × V8 (54 tissue types) for the expression of the PTPRN2 gene; Table S1: Quantitative 17-item questionnaire for Adherence to Mediterranean diet; Table S2: Taste preferences depending on the obesity status; Table S3: Association between the preference for different tastes and perception; Table S4: Rotated component matrix for the factor analysis including the five taste preferences; Table S5: Top-ranked SNPs in the GWAS for sweet taste preference (categorical variable) in the whole population (adjusted for sex, age and type 2 diabetes); Table S6: Top-ranked SNPs in the GWAS for sweet taste preference (categorical variable) in the whole population (adjusted for sex, age, type 2 diabetes and genetic PC1, PC2, PC3, PC4); Table S7: Top-ranked genes in the gene-based GWAS for sweet taste preference in the whole population; Table S8: Top-ranked SNPs in the GWAS for factor 2 in the whole population.
