# Supplementary material for: Sweet Taste Preference: Relationships with Other Tastes, Liking for Sugary Foods and Exploratory Genome-Wide Association Analysis in Subjects with Metabolic Syndrome
Source: Biomedicines. 2021 Dec 31;10(1):79. doi: 10.3390/biomedicines10010079 (PMC8772854; doi:10.3390/biomedicines10010079)
Supplement: Supplementary file 1 [file biomedicines-10-00079-s001.zip › biomedicines-1482783-supplementary.pdf]

ONLINE SUPPORTING MATERIAL

Sweet Taste Preference: Relationships with Other Tastes, Liking for Sugary Foods and Exploratory Genome-Wide Association Analysis in Subjects with Metabolic Syndrome

Rebeca Fernández-Carrión, Jose V. Sorlí, Oscar Coltell, Eva C. Pascual, Carolina Ortega-Azorín, Rocío Barragán, Ignacio M. Gimenez-Alba, Andrea Alvarez-Sala, Montserrat Fitó, Jose M Ordovas and Dolores Corella

Figure S1 2
Table S1. 3
Table S2 4
Table S3 4
Table S4 5
Figure S2 5
Table S5 6
Table S6 7
Figure S3 7
Figure S4 8
Figure S5 9
Table S7 9
Figure S6 10
Table S8 11

Figure S1. Graphic diagram representing the study design, including the sequences of experiments and statistical analysis carried out.

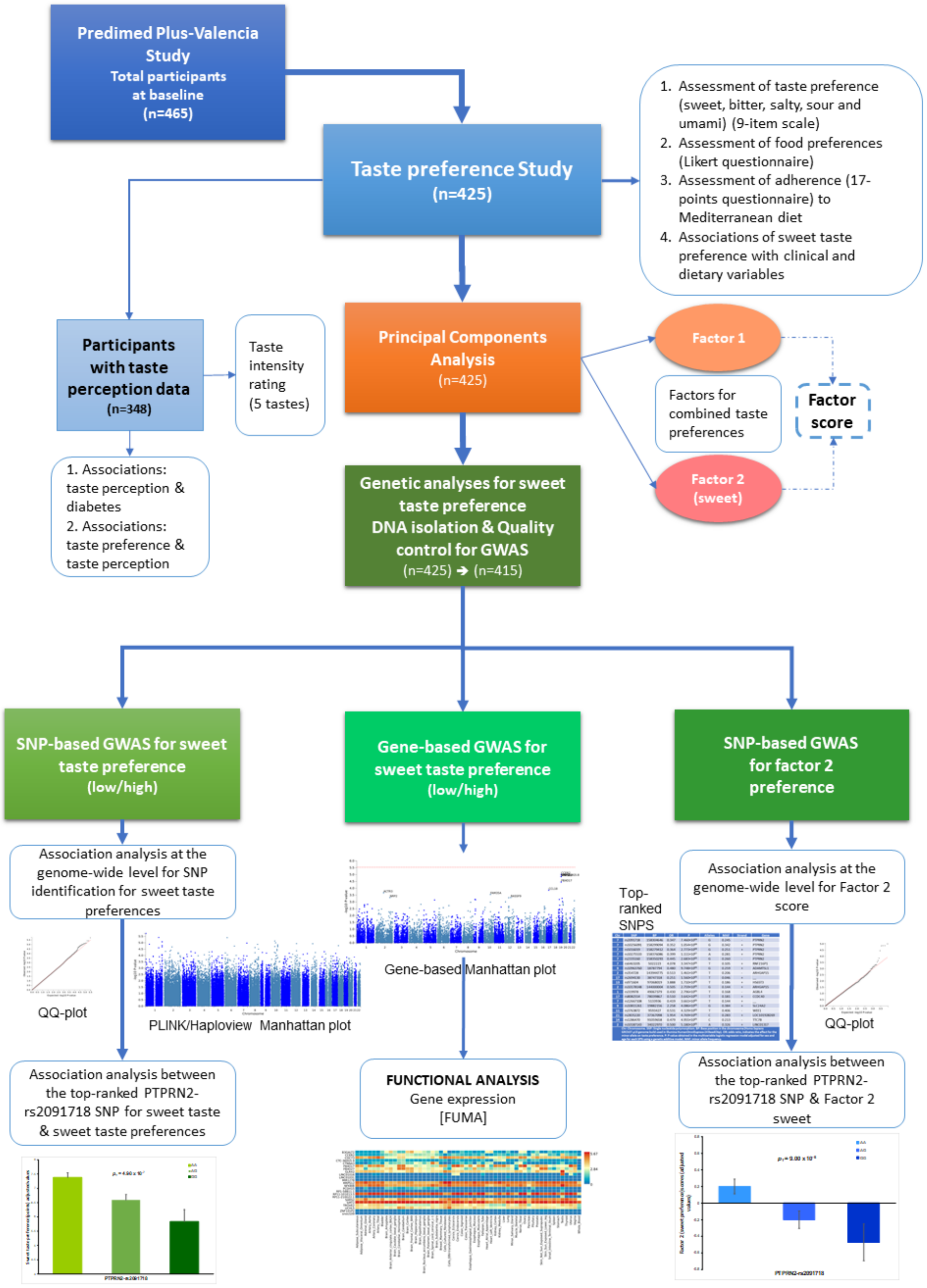

Table S1. Quantitative 17-item questionnaire of adherence to an energy-restricted Mediterranean Diet.

	Foods and frequency of consumption	Criteria for 1 point*
1.	Do you use extra virgin olive oil as the main culinary fat?	Yes
2.	How many servings of vegetables do you consume per day? (Count garnish and side servings as ½ point; 1 serving =200g)	≥2
3.	3. How many pieces of fruit or 100% natural fruit juice do you consume per day?	≥3
4.	How many servings of red meat, hamburgers, or meat products (ham, sausage, etc.) do you consume per week? (1 serving = 100-150g)	≤1
5.	How many servings of butter, margarine, or cream do you consume per week? (1 serving = 12g)	<1
6.	How many sugar-sweetened beverages (sodas, tonic waters, energy drinks, fruit juices with added sugar) do you consume per week?	<1
7.	How many servings of legumes do you consume per week? (1 serving = 150g)	≥3
8.	How many servings of fish/ shellfish do you consume per week? (1 serving = 100-150g, or 4-5 pieces of fish, or 200g of shelfish)	≥3
9.	How many times per week do you consume pastries, such as cookies, sweets or cakes?	<3
10.	How many times per week do you consume nuts[ƚ]? (1 serving = 30g)	≥3
11.	Do you preferentially consume chicken, turkey, or rabbit meat instead of beef, pork, hamburgers, or sausage?	Yes
12.	How many times per week do you consume vegetables, pasta, rice, or other dishes seasoned with *sofrito* (sauce made with tomato and onion, leek or garlic and simmered with olive oil)?	≥2
13.	Do you add sugar to beverages (coffee, tea)?	No/use artificial sweeteners
14.	How many servings of white bread do you consume per day (1 serving = 75g)?	≤1
15.	How many servings of whole grains (bread, rice, pasta) do you consume per week?	≥5
16.	How many servings of refined bread, rice and/or pasta do you consume per week?	<3
17.	17. Do you drink wine? How much do you consume per week? (1 glass = 100ml)	Men: 2-3 glasses/day<br>Women:1-2 glasses/day

*0 points if the criteria is not met.
[ƚ]including peanuts

Table S2. Taste preferences (sweet, salty, sour, umami and bitter) depending on the obesity status.

Taste preference	Obesity		
	No (n=136)	Yes (n=289)	*p*
Sweet	7.05±0.17	7.20±0.11	0.437
Salty	7.53±0.13	7.60±0.09	0.769
Sour	4.51±0.19	4.67±0.12	0.463
Umami	5.93±0.17	5.96±0.10	0.897
Bitter	4.37±0.19	4.28±0.13	0.708

Values are mean±SE; Taste preferences scores have been obtained by a 9-point hedonic scale for each taste. P-values for obesity status difference were obtained by Student's t-test.

Table S3. Association between the preference for different tastes and their perception (perceived intensity).

Taste[1]		Sweet (Sucrose) (400 mM)	Salty (NaCl) (200 mM)	Sour (Citric acid) (34 mM)	Umami (MPG) (200 mM)	Bitter (PTC) (5.6 mM)
Sweet preference	r	-0.056	0.004	-0.002	0.075	-0.050
	p	*0.297*	*0.936*	*0.976*	*0.162*	*0.356*
Salty preference	r	-0.066	-0.126	-0.105	-0.116	0.030
	p	*0.219*	*0.018*	*0.051*	*0.030*	*0.579*
Sour preference	r	-0.028	-0.110	-0.073	-0.085	0.038
	p	*0.602*	*0.040*	*0.174*	*0.115*	*0.482*
Umami preference	r	-0.034	-0.095	-0.127	-0.133	-0.055
	p	*0.531*	*0.077*	*0.018*	*0.013*	*0.309*
Bitter preference.	r	0.048	-0.105	-0.068	-0.095	-0.063
	p	*0.375*	*0.051*	*0.206*	*0.075*	*0.246*

[1]: Taste preferences scores have been obtained by a 9-point hedonic scale for each taste in the whole population. Taste perception has been obtained by rating specific tastants. Five representative tastants for the five tastes (PTC for bitter, sucrose for sweet, NaCl for salty, citric acid for sour and MPG for umami) were tested at the indicated concentrations (Concentration V). PTC: phenylthiocarbamide. MPG: L-glutamic acid monopotassium salt monohydrate. *r*: Spearman correlation coefficient. *p*: p-value for the Spearman correlation coefficient (r). n = 348 subjects with data for both taste preference and perception.

Table S4. Rotated component matrix for the factor analysis including the five taste preferences. Correlations between components and the original taste preference variables.

Taste preferences	Component	
	1	2
Sweet preference	-0.205	**0.757**
Salty preference	0.535	0.510
Sour preference	**0.832**	-0.054
Umami preference	0.104	**0.565**
Bitter preference	**0.732**	0.005

Factor analysis for the whole population (n= 425). Extraction method: Principal Component Analysis (PCA). Rotation method: Varimax with Kaiser normalization.

Figure S2. Quantile-Quantile (QQ)-plot for the SNP-based GWAS on sweet taste preference (dichotomous variable) in the whole population. Model adjusted for sex and age. Genomic Control (GC) lambda was calculated based on the 50th percentile: λ = 1.004.

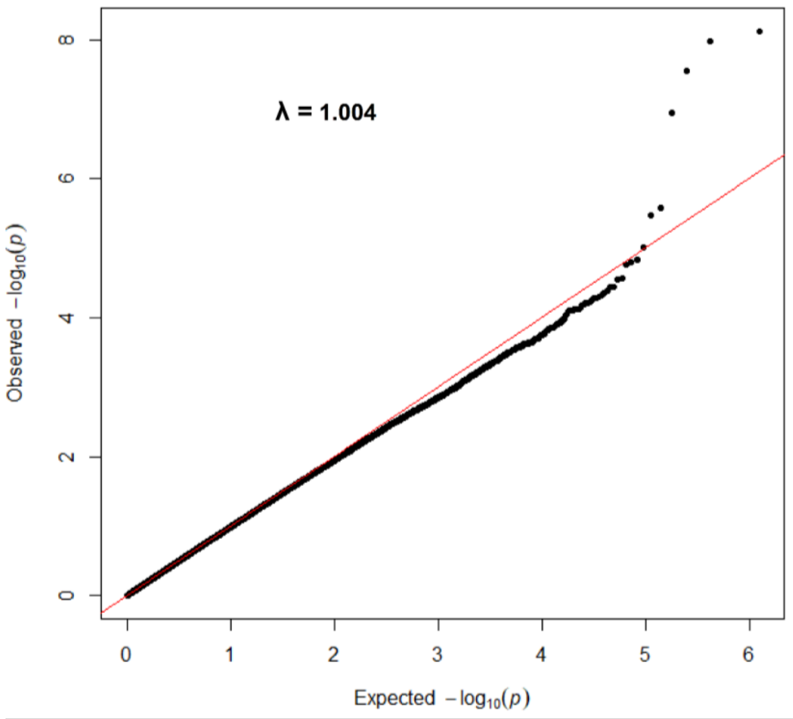

Table S5. Top-ranked SNPs in the GWAS for sweet taste preference (categorical variable) in the whole population. Model adjusted for sex, age and type 2 diabetes.

Chr	SNP	BP	OR	*p*	Alleles	MAF	Strand	Gene
7	rs2091718	158304646	0.357	$2.282x10^{-08}$	G	0.245	-	PTPRN2
7	rs10256091	158299094	0.362	$3.299x10^{-08}$	G	0.342	+	PTPRN2
7	rs5016019	158279412	0.374	$8.312x10^{-08}$	G	0.251	+	PTPRN2
7	rs10275533	158376086	0.406	$2.355x10^{-07}$	A	0.281	+	PTPRN2
7	rs6463205	5022223	4.080	$3.784x10^{-06}$	T	0.105	+	RNF216P1
7	rs2335160	158350293	0.454	$5.349x10^{-06}$	G	0.260	-	PTPRN2
9	rs10963760	18787794	0.479	$1.053x10^{-05}$	G	0.259	+	ADAMTSL1
13	rs971604	97068019	3.938	$1.703x10^{-05}$	T	0.186	+	HS6ST3
17	rs2694130	38747318	0.257	$2.496x10^{-05}$	T	0.046	+	intergenic
11	rs3763872	9593427	0.518	$2.701x10^{-05}$	T	0.406	-	WEE1
21	rs2835220	37367098	2.002	$3.190x10^{-05}$	C	0.283	+	LOC101928269
7	rs12667108	5133936	0.415	$3.553x10^{-05}$	T	0.144	+	intergenic
21	rs762364	37406849	2.100	$3.622x10^{-05}$	G	0.255	+	SETD4
2	rs354728	143944775	0.527	$3.726x10^{-05}$	T	0.206	-	ARHGAP15
14	rs11157364	44236516	0.536	$3.789x10^{-05}$	C	0.445	+	intergenic
14	rs1957280	44232069	0.540	$3.808x10^{-05}$	T	0.444	+	intergenic
10	rs10826596	29585987	2.354	$4.005x10^{-05}$	G	0.255	+	LYZL1
12	rs7301498	94988487	0.524	$4.700x10^{-05}$	A	0.362	+	TMCC3
14	rs1286470	91059658	0.478	$5.289x10^{-05}$	C	0.213	-	TTC7B
1	rs319978	49067379	0.442	$5.460x10^{-05}$	T	0.168	-	AGBL4
13	rs9562605	32890026	0.510	$5.765x10^{-05}$	T	0.226	+	BRCA2
2	rs10187143	34022970	0.501	$5.957x10^{-05}$	A	0.326	+	LINC01317
7	rs6961543	5120148	2.337	$5.987x10^{-05}$	A	0.207	+	intergenic
21	rs2835223	37369811	0.543	$6.397x10^{-05}$	G	0.389	+	LOC101928269
2	rs10178148	144000004	0.519	$6.674x10^{-05}$	G	0.144	+	ARHGAP15
6	rs9490525	123018092	0.516	$6.797x10^{-05}$	A	0.148	+	PKIB
9	rs10811261	19882156	2.206	$6.964x10^{-05}$	G	0.384	+	SLC24A2
3	rs2639256	81052987	1.866	$7.109x10^{-05}$	A	0.388	-	LINC02027
2	rs2556094	160685529	2.706	$7.426x10^{-05}$	A	0.207	+	LY75-CD302
17	rs8082554	78039867	0.523	$7.545x10^{-05}$	T	0.181	+	CCDC40
17	rs12451399	43140100	0.462	$7.916x10^{-05}$	T	0.149	+	DCAKD
17	rs12449933	43137201	0.463	$8.176x10^{-05}$	T	0.149	+	DCAKD
3	rs17391287	80792633	1.966	$8.248x10^{-05}$	C	0.271	+	LOC105377177
13	rs238256	42923675	1.906	$8.673x10^{-05}$	G	0.441	-	intergenic
18	rs4797396	10011664	0.474	$8.888x10^{-05}$	T	0.196	+	intergenic
21	rs2212917	37381848	0.426	$9.137x10^{-05}$	A	0.178	+	intergenic
16	rs7192533	60161902	0.543	$9.828x10^{-05}$	T	0.378	+	intergenic
18	rs4799085	77533722	0.283	$1.010x10^{-04}$	T	0.168	+	intergenic

Chr: Chromosome. SNP: Single nucleotide polymorphism. BP: Base position in the chromosome (Homo Sapiens GRCh37.p13 genome build used in Illumina HumanOmniExpress-24 BeadChip). OR: odds ratio, indicates the effect for the minor allele on sweet taste preference. P: P-value obtained in the multivariable logistic regression model adjusted for sex, age and type 2 diabetes for each SNP using a genetic additive model. MAF: minor allele frequency.

Table S6. Top-ranked SNPs in the GWAS for sweet taste preference (categorical variable) in the whole population. Model adjusted for sex, age, type 2 diabetes and the four highest PCA eigenvalues principal components PC1, PC2, PC3 and PC4.

CHR	SNP	BP	OR	*P*	Alleles	MAF	Strand	Gene
7	rs2091718	158304646	0.344	$7.206x10^{-09}$	G	0.245	-	PTPRN2
7	rs10256091	158299094	0.348	$1.015x10^{-08}$	G	0.342	+	PTPRN2
7	rs5016019	158279412	0.360	$2.658x10^{-08}$	G	0.251	+	PTPRN2
7	rs10275533	158376086	0.397	$1.021x10^{-07}$	A	0.281	+	PTPRN2
7	rs2335160	158350293	0.440	$2.244x10^{-06}$	G	0.260	-	PTPRN2
7	rs6463205	5022223	4.199	$3.293x10^{-06}$	T	0.105	+	RNF216P1
2	rs354728	143944775	0.507	$1.108x10^{-05}$	T	0.206	-	ARHGAP15
9	rs10963760	18787794	0.485	$1.348x10^{-05}$	G	0.259	+	ADAMTSL1
12	rs7301498	94988487	0.508	$2.248x10^{-05}$	A	0.362	+	TMCC3
17	rs2694130	38747318	0.245	$2.500x10^{-05}$	T	0.046	+	intergenic
2	rs10178148	144000004	0.503	$2.565x10^{-05}$	G	0.144	+	ARHGAP15
7	rs12667108	5133936	0.411	$2.706x10^{-05}$	T	0.144	+	intergenic
11	rs3763872	9593427	0.523	$3.021x10^{-05}$	T	0.406	-	WEE1
13	rs971604	97068019	3.799	$3.153x10^{-05}$	T	0.186	+	HS6ST3
9	rs10811261	19882156	2.302	$3.236x10^{-05}$	G	0.384	+	SLC24A2
14	rs1286470	91059658	0.469	$3.608x10^{-05}$	C	0.214	-	TTC7B
5	rs6890147	20685297	0.513	$4.374x10^{-05}$	G	0.409	+	LOC105374676
7	rs6961543	5120148	2.389	$4.933x10^{-05}$	A	0.207	+	intergenic
17	rs8082554	78039867	0.517	$5.295x10^{-05}$	T	0.181	+	CCDC40
1	rs319978	49067379	0.441	$5.533x10^{-05}$	T	0.168	-	AGBL4
10	rs10826596	29585987	2.305	$5.798x10^{-05}$	G	0.255	+	LYZL1

PCA: Principal Component Analysis. Chr: Chromosome. SNP: Single nucleotide polymorphism. BP: Base position in the chromosome (Homo Sapiens GRCh37.p13 genome build used in Illumina HumanOmniExpress-24 BeadChip). OR: odds ratio, indicates the effect for the minor allele on taste preference. P: *P*-value obtained in the multivariable logistic regression model adjusted for sex, age, type 2 diabetes and for the four main principal components (PCs) eigenvalues obtained from a PCA where a total of 20 PCs were computed. MAF: minor allele frequency.

Figure S3. QQ-plot for the SNP-based GWAS on sweet taste preference (dichotomous variable) in the whole population. Model adjusted for sex, age, diabetes, and principal components PC1, PC2, PC3 and PC4. Genomic Control (GC) lambda was calculated based on the 50th percentile and was 1.022.

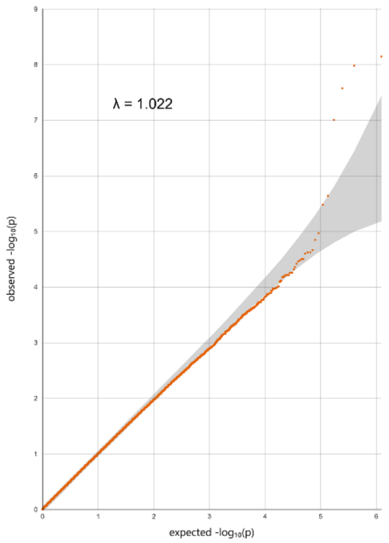

Figure S4. Regional plot for the lead SNP PTPRN2-rs2091718, on chromosome 7. Each SNP are colored based on r^2. Combined Annotation Dependent Depletion (CADD) score, as well as the RegulomeDB score, are shown. RegulomeDB score is a categorical score from 1a to 7: *1a* means that those SNPs are most likely affecting regulatory elements [65].

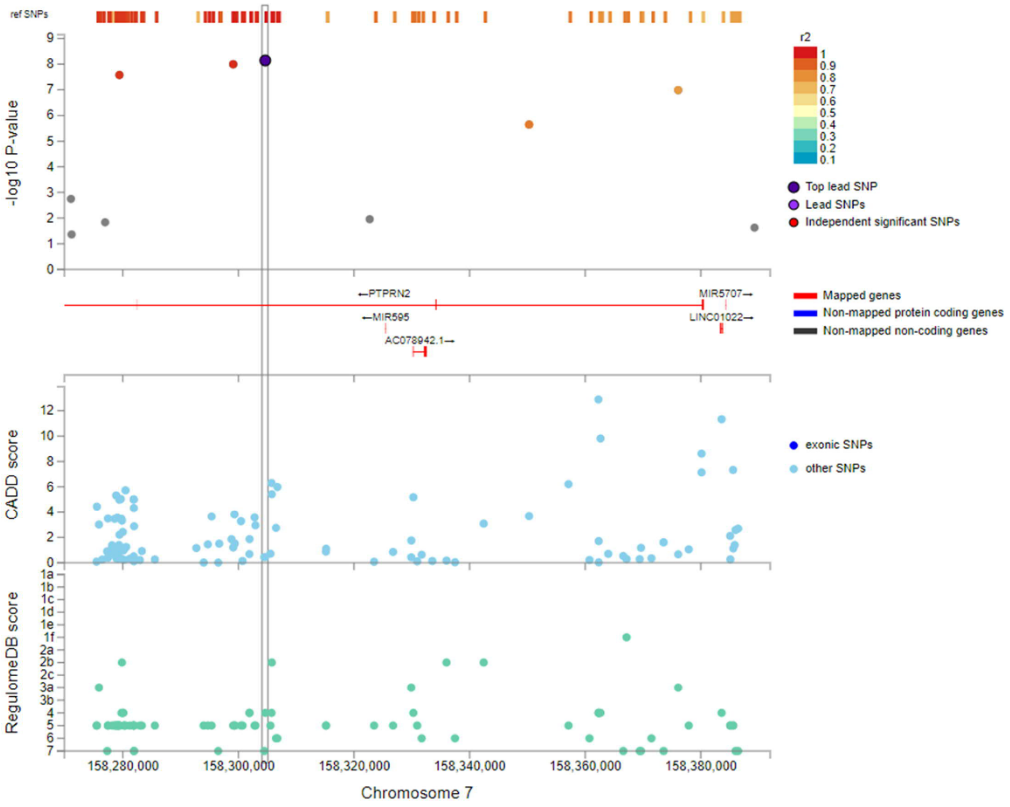

No eQTL of selected tissues exists in this region.

Regulome DB

RegulomeDB Categorical Scores

Category	Description
	Likely to affect binding and linked to expression of a gene target
1a	eQTL + TF binding + matched TF motif + matched DNase footprint + DNase peak
1b	eQTL + TF binding + any motif + DNase footprint + DNase peak
1c	eQTL + TF binding + matched TF motif + DNase peak
1d	eQTL + TF binding + any motif + DNase peak
1e	eQTL + TF binding + matched TF motif
1f	eQTL + TF binding/DNase peak
	Likely to affect binding
2a	TF binding + matched TF motif + matched DNase footprint + DNase peak
2b	TF binding + any motif + DNase footprint + DNase peak
2c	TF binding + matched TF motif + DNase peak
	Less likely to affect binding
3a	TF binding + any motif + DNase peak
3b	TF binding + matched TF motif
	Minimal binding evidence
4	TF binding + DNase peak
5	TF binding or DNase peak
6	Motif hit
	No binding evidence
7	No evidence
NA	the variant does not exist in RegulomeDB

Figure S5. Q-Q plot for the gene-based GWAS on the sweet taste preference (adjusted for sex and age), obtained using FUMA [65].

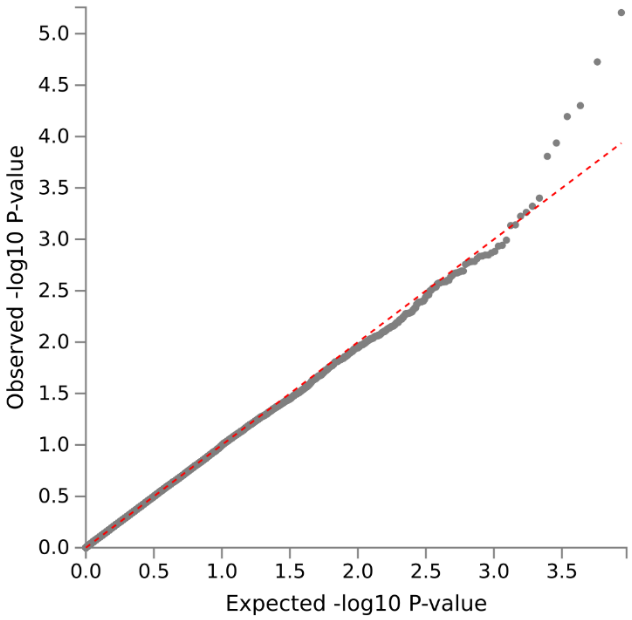

Table S7. Top-ranked genes in the gene-based GWAS using MAGMA [65,71] and computing the gene level p-value for sweet taste preference in the whole population.

Symbol	Gene (ENSEMBL ID)	Chr	Start	Stop	Size (bp)	*p*
AP000688.1	ENSG00000268098	21	37402368	37417818	15450	$6.229x10^{-06}$
PTPRN2	ENSG00000155093	7	157331750	158380480	1048730	$1.878x10^{-05}$
SETD4	ENSG00000185917	21	37406839	37451687	44848	$5.007x10^{-05}$
LYZL1	ENSG00000120563	10	29577990	29607257	29267	$6.385x10^{-05}$
CLDN20	ENSG00000171217	6	155585147	155597682	12535	$1.156x10^{-04}$
LHB	ENSG00000104826	19	49519237	49520338	1101	$1.558x10^{-04}$
CCDC40	ENSG00000141519	17	78010435	78074412	63977	$3.966x10^{-04}$
GAA	ENSG00000171298	17	78075355	78093678	18323	$4.766x10^{-04}$
INSIG1	ENSG00000186480	7	155089486	155101945	12459	$5.471x10^{-04}$
ARMC1	ENSG00000104442	8	66514694	66546442	31748	$5.964x10^{-04}$
ABCB9	ENSG00000150967	12	123405498	123466196	60698	$7.233x10^{-04}$
PDE11A	ENSG00000128655	2	178487980	178973066	485086	$7.334x10^{-04}$

CHR: Chromosome. START: Base position in the chromosome (Homo Sapiens GRCh38 genome build) where the gene starts. STOP: Base position in the chromosome (Homo Sapiens GRCh38 genome build) where the gene ends. SIZE (bp): size of the gene in base pairs (bp), calculated as STOP - START. P: *P*-values obtained by MAGMA in the gene-set analysis adjusted for sex and age. For statistical significance, 2.7×10^{-6} is considered the level of significant evidence at the gene level analysis. Likewise, $p<1 \times 10^{-4}$ is considered as the suggestive level of gene-based association.

Figure S6. Gene expression heat map based on the data set GTEx V8 (54 tissue types) (average expression per label) for the expression of the PTPRN2 gene and three top-ranked genes obtained from the gene-based GWAS on sweet taste preference (dichotomous variable). We used the FUMA-GENE2FUNC tool [65].

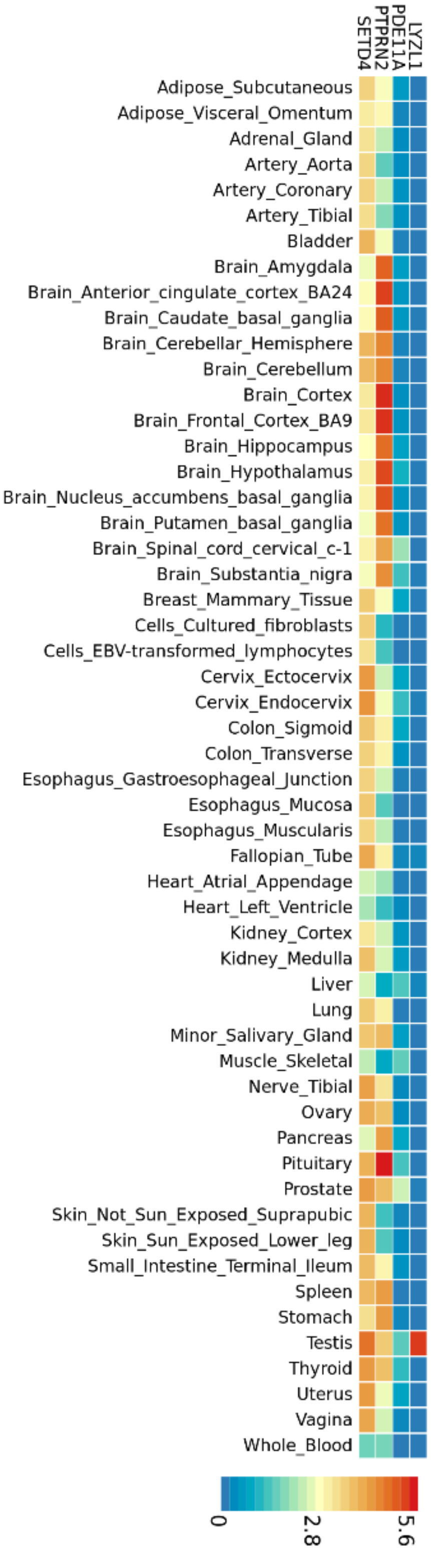

Table S8. Top-ranked SNPs in the GWAS for factor 2 (labeled as 'sweet/umami taste') in the whole population. Additive model adjusted by sex and age.

Chr	SNP	BP	Beta	*p*	Alleles	MAF	Strand	Gene
17	rs2694130	38747318	-0.728	$9.647x10^{-07}$	T	0.0459	+	intergenic
18	rs4799085	77533722	-0.738	$9.952x10^{-07}$	T	0.1675	+	intergenic
9	rs4484780	88860820	0.348	$3.126x10^{-06}$	C	0.3257	+	C9orf153
9	rs10118760	88854546	0.349	$3.676x10^{-06}$	T	0.3129	+	C9orf153
3	rs7642458	80859757	0.323	$4.092x10^{-06}$	A	0.4177	+	intergenic
3	rs17391287	80792633	0.335	$6.769x10^{-06}$	C	0.2708	+	LOC105377177
9	rs7848944	88859144	0.335	$7.495x10^{-06}$	C	0.4022	+	C9orf153
7	rs1229670	26447021	-0.922	$7.861x10^{-06}$	G	0.1416	+	LOC107983953
16	rs237142	26646032	0.317	$8.770x10^{-06}$	A	0.4411	+	intergenic
7	rs5016019	158279412	-0.369	$9.156x10^{-06}$	G	0.2512	+	PTPRN2
9	rs2224954	17771828	0.317	$9.471x10^{-06}$	C	0.4639	+	SH3GL2
9	rs4302941	17772909	0.310	$1.108x10^{-05}$	A	0.4609	+	SH3GL2
20	rs394732	52675188	-0.319	$1.257x10^{-05}$	G	0.4239	+	BCAS1
8	rs13278035	49509249	-0.651	$1.265x10^{-05}$	G	0.0627	+	LOC101929268
13	rs7323695	102113468	-1.236	$1.275x10^{-05}$	A	0.1192	+	ITGBL1
2	rs2136492	221704949	-0.737	$1.334x10^{-05}$	C	0.0078	-	LOC107985988
3	rs10511108	80714827	0.328	$1.405x10^{-05}$	C	0.2310	-	LOC105377177
14	rs8020310	74494437	-0.482	$1.417x10^{-05}$	G	0.1548	+	BBOF1
19	rs10221473	7236626	-0.304	$1.439x10^{-05}$	A	0.4884	+	INSR
5	rs2569235	171897090	0.308	$1.475x10^{-05}$	C	0.3564	-	intergenic
10	rs10902892	124949389	-1.553	$1.489x10^{-05}$	C	0.2200	+	LOC107984275
7	rs10275533	158376086	-0.343	$1.647x10^{-05}$	A	0.2810	+	PTPRN2
5	rs4091221	163713876	-0.320	$2.006x10^{-05}$	T	0.1697	+	intergenic
7	rs2091718	158304646	-0.355	$2.007x10^{-05}$	G	0.2454	-	PTPRN2
8	rs4733210	30816027	0.493	$2.181x10^{-05}$	G	0.3125	+	intergenic
3	rs13086426	80839513	0.320	$2.239x10^{-05}$	A	0.2312	+	intergenic
9	rs1886589	17761379	-0.292	$2.348x10^{-05}$	C	0.4549	+	SH3GL2
18	rs3018270	47447780	-0.316	$2.478x10^{-05}$	C	0.4305	+	MYO5B
7	rs10256091	158299094	-0.351	$2.523x10^{-05}$	G	0.3421	+	PTPRN2

Chr: Chromosome. SNP: Single nucleotide polymorphism. BP: Base position in the chromosome (Homo Sapiens GRCh37.p13 genome build used in Illumina HumanOmniExpress-24 BeadChip). BETA: indicates the effect for the minor allele on the Factor 2 variable for sweet taste preference. P: *P*-value obtained in the multivariable general linear regression model adjusted for sex and age using a genetic additive model. MAF: minor allele frequency.
